# An Information-Theoretic Approach to Quantitative Analysis of the Correspondence Between Skin Blood Flow and Functional Near-Infrared Spectroscopy Measurement in Prefrontal Cortex Activity

**DOI:** 10.3389/fnins.2019.00079

**Published:** 2019-02-15

**Authors:** Soheil Keshmiri, Hidenobu Sumioka, Masataka Okubo, Hiroshi Ishiguro

**Affiliations:** ^1^Hiroshi Ishiguro Laboratories, Advanced Telecommunications Research Institute International, Kyoto, Japan; ^2^Graduate School of Engineering Science, Osaka University, Osaka, Japan

**Keywords:** functional near-infrared spectroscopy, skin blow flow, transfer entropy, conditional entropy, frontal cortex activity

## Abstract

Effect of Skin blood flow (SBF) on functional near-infrared spectroscopy (fNIRS) measurement of cortical activity proves to be an illusive subject matter with divided stances in the neuroscientific literature on its extent. Whereas, some reports on its non-significant influence on fNIRS time series of cortical activity, others consider its impact misleading, even detrimental, in analysis of the brain activity as measured by fNIRS. This situation is further escalated by the fact that almost all analytical studies are based on comparison with functional Magnetic Resonance Imaging (fMRI). In this article, we pinpoint the lack of perspective in previous studies on preservation of information content of resulting fNIRS time series once the SBF is attenuated. In doing so, we propose information-theoretic criteria to quantify the necessary and sufficient conditions for SBF attenuation such that the information content of frontal brain activity in resulting fNIRS times series is preserved. We verify these criteria through evaluation of their utility in comparative analysis of principal component (PCA) and independent component (ICA) SBF attenuation algorithms. Our contributions are 2-fold. First, we show that mere reduction of SBF influence on fNIRS time series of frontal activity is insufficient to warrant preservation of cortical activity information. Second, we empirically justify a higher fidelity of PCA-based algorithm in preservation of the fontal activity's information content in comparison with ICA-based approach. Our results suggest that combination of the first two principal components of PCA-based algorithm results in most efficient SBF attenuation while preserving maximum frontal activity's information. These results contribute to the field by presenting a systematic approach to quantification of the SBF as an interfering process during fNIRS measurement, thereby drawing an informed conclusion on this debate. Furthermore, they provide evidence for a reliable choice among existing SBF attenuation algorithms and their inconclusive number of components, thereby ensuring minimum loss of cortical information during SBF attenuation process.

## 1. Introduction

Functional near-infrared spectroscopy (fNIRS) time series of brain cortical activity is subject to various systemic physiological interferences, ranging from cardiac activity and respiration (Orbig et al., 2000; Tonorov et al., 2000; Payne et al., 2003) to motion artifacts (Cooper et al., 2012; Tak and Ye, 2014; Naseer and Hong, 2015) and scalp-hemodynamic (Scholkmann et al., 2014). While detection and correction strategies for most of these physiological and systemic interferences are widely practiced, effect of scalp-hemodynamic and skin blood flow (SBF) has been subject to much divided perspective. Whereas, Takahashi et al. (2011) argued that a major part of task-evoked changes in blood oxygenation hemoglobins (ΔOxy-Hb) in the forehead is due to SBF, Sato et al. (2013) claimed that such changes are highly correlated with blood oxygen level dependent (BOLD) of functional magnetic resonance imaging (fMRI) and gray matter, as opposed to SBF or other soft tissues. This, in part, helps explain the lack of incorporation of analytical steps for SBF attenuation in most recent studies (Liand et al., 2010; Cuiand et al., 2011; Gagnonand et al., 2012; Fishbum et al., 2014; Ozawa et al., 2014; Bakerand et al., 2016; Guand et al., 2017; Liu et al., 2017) as well as NIRS-related analytical toolkits (Koh et al., 2007; Huppertand et al., 2009; Strangmann, 2009; Ye et al., 2009; Feketeand et al., 2011).

However, research suggests that systemic changes due to SBF are unpreventable since they are the result of the activation of the autonomic nervous system and/or varying blood pressure in response to action (Lee et al., 2002; Scremin and Kenney, 2004). In fact, Sato et al. (2016) argued that scalp- and cerebral-hemodynamic (in particular ΔOxy-Hb) increase in a task-related manner, implying their similar temporal profiles. In light of these observations, the field has witnessed a growing number of research on SBF attenuation approaches. One class of SBF filters has considered the direct measurements from short source-detector distances (e.g., ≤ 2.0 cm) to attenuate the effect of scalp-hemodynamic on channels with longer distance (e.g., 3.0 *cm* ≤ *d* ≤ 4.5 *cm*) (Zhang et al., 2009; Gagnon et al., 2011, 2012, 2014; Saager et al., 2011; Haeussinger et al., 2014), where *d* stands for source-detector distance. Another family of such filters has been built upon the assumption that scalp-hemodynamic changes are more global than cerebral-hemodynamic, thereby introducing mathematically founded techniques for SBF removal. For instance, Zhang et al. (2005) assumed the orthogonality between the spatial interference and the spatial evoked activation subspaces to derive principal components from resting periods, thereby eliminating such components from task period as representatives of SBF. This approach was further extended by Zhang et al. (2016) through application of Gaussian filtering. On the other hand, Kohno et al. (2007) employed independent component analysis (ICA) to extract the most spatially uniform component of ΔOxy-Hb. Furthermore, they showed their extracted component was highly correlated with SBF through its comparison with simultaneous laser Doppler measurement (Johnson et al., 1984; Oberg, 1990). Kiguchi and Funane (2014) further extended this ICA-based approach for its online use. Sato et al. (2016) argued that although these filters offer reliable and accurate tool for SBF attenuation, their reliance on considerably large number of probes for data acquisition makes their application infeasible. In turn, they addressed this limitation through identification of the scalp-hemodynamic component from a small number of short source-detector channels, thereby removing this effect based on general linear model (GLM) (Mardia et al., 1979; Friston et al., 1995, 2011). Their approach showed a significant improvement in contrast with Zhang et al. (2005) and Kohno et al. (2007), as suggested by their fMRI-based comparative analysis.

Although our overview of research on scalp-hemodynamic and SBF demonstrates fascinating approaches with impressive results, it reveals an immediate shortcoming that is lack of quantitative realization of the utility of these methodologies for their verification toward a common consensus on their use. For instance, whereas Zhang et al. (2005) claimed the first three principal components for adequate SBF attenuation, Sato et al. (2016) suggested a non-significant difference in adaptation of first or combination of first two or three components. More importantly, these approaches fall short in assessing the state of fNIRS time series once the SBF attenuation process is complete. For example, Kohno et al. (2007) showed a high correlation between their selected most spatially uniform component with SBF signal while discarding a report on fNIRS content once this component was removed. Although a few have included measures such as correlation coefficient, signal-to-noise ratio, or Pearson *R*^2^ (Zhang et al., 2005; Gagnon et al., 2011), these linear measures fail to detect the nonlinearity in interacting processes (Kinney and Atwal, 2014). Moreover, they cannot provide any causal insight on the observed effect: they are unable to quantify whether they attenuated the confounding effect of the interfering process or information pertinent to cortical activity. In addition, many of these results have derived the quality of their SBF attenuation through comparison of fNIRS data with fMRI recordings (Haeussinger et al., 2014; Sato et al., 2016). Although high spatial resolution of fMRI along with significant correlation between its BOLD and corresponding Blood de-/Oxygenation of fNIRS (Okamoto et al., 2004; Steinbrink et al., 2006; Strangman et al., 2006; Toronov et al., 2007; Cuiand et al., 2011) provide a basis for such comparative analyses, they are highly time-consuming and require extra care to ensure the least environmental and experimental discrepancies, making their adaptation impractical in a broader domain.

In this article, we address these shortcomings through proposal of information-theoretic criteria for preservation of information content of frontal brain activity. We utilize the concept of transfer entropy (TE) (Schreiber, 2000) to quantify the effect of SBF on fNIRS time series of frontal brain activity via transferring undesired information onto fNIRS measurement. TE provides a powerful tool for measuring the strength and direction (i.e., causation) of the coupling between simultaneously observed processes (Kaiser and Schreiber, 2002). Utilization of TE as a measure for information flow becomes more attractive, considering its minimal assumptions on dynamics of the time series under investigation, its numerical stability even for reasonably small sample sizes, and its ability in capturing both, linear as well as nonlinear effects (Lungarella and Sporns, 2006). In fact, recent years have witnessed a growing interest in application of TE in neuroscientific research (Lungarella and Sporns, 2006; Honey et al., 2007; Vakorin et al., 2010; Liao et al., 2011). Additionally, we exploit the concept of mutual information (MI) and its correspondence with conditional entropy between interacting continuous random variables (Cover and Thomas, 2006; Stone, 2015) to formalize criteria for preservation of the frontal activity's information in resulting fNIRS time series once the SBF attenuation is complete.

Our results suggest that mere reduction of SBF influence on fNIRS time series of frontal activity is insufficient to warrant frontal activity's information is retained. Moreover, they imply a higher fidelity of PCA-based algorithm in frontal activity's information preservation in comparison with ICA-based approach. Furthermore, they indicate that a combination of first two principal components of PCA-based algorithm results in most efficient SBF attenuation while ensuring maximum frontal activity's information preservation. Our results contribute to the field by presenting a systematic approach to quantification of the SBF and its effect as an interfering process during fNIRS measurement, thereby drawing an informed conclusion on this debate. Furthermore, they provide evidence for a reliable choice among existing SBF attenuation algorithms and their inconclusive number of components, thereby ensuring minimum loss of frontal activity's information during SBF attenuation process.

## 2. Materials and Methods

### 2.1. Formal Statements

#### 2.1.1. Preliminaries

Prior to formalizing our information-theoretic criteria, we restate the definitions of conditional entropy, MI, and TE, along with two Theorems and a Corollary (without proofs) from information theory to help better elaborate on rationale behind our criteria.

**Definition 2.1**. *If* (X,Y)~f(x,y), ∀x∈X,y∈Y*, the conditional entropy* H(X|Y) *is defined as Cover and Thomas (**2006**)[p. 249]*

(1) H(X|Y)=−∫f(x,y)log(f(x|y))dxdy

where f(x,y), ∀x∈X,y∈Y indicates the joint density and *f*(*x*|*y*) is the probability of occurrence of *x*, given that *y* occurred. In other words, conditional entropy quantifies the average uncertainty regarding the value of X when the value of Y is known (Stone, 2015).

**Definition 2.2**. *The mutual information* I(X;Y) *between two random variables with joint density* f(x,y), ∀x∈X,y∈Y *is defined as Cover and Thomas (**2006**)[p. 251]*

(2) $$I(X;Y)=\int f(x,y)\log\frac{f(x,y)}{f(x)f(y)}dxdy$$

Mutual information can be expressed in terms of conditional entropy as (ibid.):

(3) $$\begin{array}{l} I(X;Y)=H(X)-H(X|Y)\\ \;=H(Y)-H(Y|X)\\ \;=H(X)+H(Y)-H(X,Y) \end{array}$$

with *H*(.) representing the entropy of its argument and H(X|Y) and H(X,Y) are the conditional entropy and the joint entropy between X and Y, respectively.

On the other hand, transfer entropy (Schreiber, 2000) aims at extracting directed flow or transfer of information (Lungarella and Sporns, 2006) between interacting processes. In essence, TE quantifies the deviation from generalized Markov property $p\left(x_{t+1}|x_{t},y_{t}\right)=p\left(x_{t+1}|x_{t}\right),\;\forall x_{t},x_{t+1}\in X,y_{t}\in Y$, with *p*(*x*|*y*) being the probability of occurrence of *x*, given *y* occurred. TE is expressed as a specific version of Kullback-Leibler divergence (Cover and Thomas, 2006; Stone, 2015) i.e., the relative entropy (Lungarella and Sporns, 2006):

(4) $$T(Y\to X)=\sum_{x_{t+1}}\sum_{x_{t}}\sum_{y_{t}}p(x_{t+1},x_{t},y_{t})\log\frac{p(x_{t+1}|x_{t},y_{t})}{p(x_{t+1}|x_{t})}$$

One can also identify TE as a conditional MI (i.e., a causal inference on shared information) between two interacting processes:

(5) $$T(Y\to X)=I(y_{t};x_{t+1}|x_{t}),\;\forall x_{t+1},x_{t}\in X,\;y_{t}\in Y$$

If this deviation is small, then the state of Y is assumed to have minimal or no relevance on the transition probabilities of X (Lungarella and Sporns, 2006), thereby implying an absence and/or a non-significant effect of Y on X. It is worthy of note that unlike MI, TE is explicitly and strictly non-symmetric under exchange of the role of the interacting processes (Kaiser and Schreiber, 2002). In other words, TE(Y→X)≠TE(X→Y),∀X, Y.

**Theorem2.1**. *(Conditioning reduces entropy): For any two random variables* X *and* Y*, we have (Cover and Thomas*, *2006**, p. 41 and p. 253)*

(6) H(X|Y)≤H(X)

*with equality if and only if* X *and* Y *are independent*.

**Theorem 2.2**. *(Data Processing Inequality): if* X→Y→Z^1^*, then (Cover and Thomas*, *2006**, p. 34)*

(7) I(X;Y)≥I(X;Z)

**Corollary 2.1**. *In particular, if* Z=g(Y)*, we have (Cover and Thomas*, *2006**, p. 35)*

(8) I(X;Y)≥I(X;Y)

Concretely, Data Processing Inequality (DPI), as formulated in Equations (7, 8), states that the result of any manipulation of data cannot improve the inferences that are made from the data (Cover and Thomas, 2006; Kinney and Atwal, 2014). In other words, no matter how sophisticated a processing approach is, it inevitably results in loss of information. Implication of DPI in SBF attenuation is 2-fold: (1) it implies that no matter how well-defined an SBF attenuation process is, loss of information is inherent in its steps. (2) Therefore, it is of utmost cruciality to ensure such a loss maximally reflects the undesirable information induced by SBF than actual frontal brain activity.

In what follows, we utilize the aforementioned observations from information theory to formalize our criteria. In essence, Criterion 2.1 through Criterion 2.3 provide necessary and sufficient conditions for quantification of the ability of SBF attenuation algorithms in reducing effect of SBF on fNIRS time series of frontal brain activity. On the other hand, Criterion 2.4 signifies utility of such algorithms in preservation of the frontal brain activity's information in resulting fNIRS times series. Finally, Criterion 2.5 examines whether preservation of the cortical activity in resulting fNIRS time series is maximized by adapted SBF algorithms.

#### 2.1.2. The Criteria

Let X and *X*′ represent the fNIRS time series of cortical activity before and after application of an SBF attenuation algorithm. Furthermore, let Y be the time series, representing SBF. The main premise of any SBF attenuation algorithm is its effectiveness for reducing the impact of SBF on fNIRS time series of cortical activity of human subjects.

**Criterion 2.1**. *Transferred information from SBF to fNIRS times series is significantly reduced if adapted attenuation process is effective i.e.*, *TE*(*Y* → *X*′) ≤ *TE*(*Y* → *X*).

Criterion 2.1, in turn, implies that given different SBF attenuation algorithms, the one with significantly smaller *TE*(*Y* → *X*′) is more effective in attenuation of SBF influence on fNIRS time series of frontal brain activity in comparison with other SBF attenuation algorithms.

Furthermore, if *X* primarily represents the frontal activity that is partially contaminated by SBF, then attenuation of SBF must, in principle, have no effect on ability of *X* to explain *X*′ in absence of *Y*. This, in turn, implies that *Y* → *X* → *X*′ holds true (as per Theorem 2.2) and *X*′ = *g*(*X*) i.e., *X*′ is expressible as a function of X. We utilize this observation in conjunction with Corollary 2.1 to formalize our second criterion.

**Criterion 2.2**. *fNIRS time series prior to SBF attenuation must, in principle, have more in common with Y than after SBF attenuation is complete i.e.*, *I*(*Y*; *X*) ≥ *I*(*Y*; *g*(*X*))*. Using Theorem* *2.2* *and applying Equation (3), we get*

(9) $$\begin{array}{l} \;I(Y;X)\geq I(Y;X^{\prime })\\ \Rightarrow H(Y)-H(Y|X)\geq H(Y)-H(Y|X^{\prime })\\ \;\Rightarrow H(Y|X)\leq H(Y|X^{\prime }) \end{array}$$

As a direct consequence of Criterion 2.2, an SBF attenuation algorithm with significantly larger *H*(*Y*|*X*′) is more effective, in comparison with any other such algorithms.

Criterion 2.1 and Criterion 2.2 provide necessary conditions to validate that outcome of an adapted SBF attenuation algorithm is successful in reducing the correspondence between SBF and fNIRS time series of frontal brain activity. However, they are not sufficient conditions for quantification of significance of such a reduction since *H*(*Y*|*X*′) ≠ *H*(*X*′|*Y*), ∀*X*′, *Y*. Therefore, it is necessary to validate the sufficient condition of such an algorithm in reducing the SBF effect.

**Criterion 2.3**. *Y must, in principle, be more informative about fNIRS before than after attenuation is complete i.e.*, *H*(*X*|*Y*) ≤ *H*(*X*′|*Y*)

As a result of Criterion 2.3, if an SBF attenuation algorithm is more effective, its application must result in significantly larger *H*(*X*′|*Y*) in comparison with other such algorithms.

Moreover, an effective SBF attenuation algorithm ensures the preservation of cortical activity while reducing the undesirable effect of SBF, thereby resulting in higher mutual information between X and *X*′ than X and Y. We formulate this expectation through the fourth criterion.

**Criterion 2.4**. *X*′ *realizes the information content of* *X* *more than* *Y* *does i.e.*, *I*(*X*; *X*′) ≥ *I*(*X*; *Y*)*. Applying Equation (3), this is equivalent to*

(10) $$\begin{array}{l} H(X)-H(X|X^{\prime })\geq H(X)-H(X|Y)\\ \;\Rightarrow H(X|X^{\prime })\leq H(X|Y) \end{array}$$

Considering Criterion 2.4, an effective SBF attenuation algorithm results in significantly smaller *H*(*X*|*X*′) in comparison with other SBF attenuation algorithms. Criterion 2.4 forms a necessary condition to ensure that the acquired time series is primarily due to cortical activity that is affected by SBF than vice-versa, as demonstrated through the following Theorem.

**Theorem 2.3**. *Let X*, *C, and Y represent recorded fNIRS, actual cortical activity, and SBF such that X* = *C* + *Y. Then, Criterion* *2.4* *is a necessary condition if* *C* *is reflective of cortical activity*.

*Proof:* Taking derivative of X = C + Y with respect to C (i.e., variable of interest), we have:

(11) $$\frac{dX}{dC}=\frac{d(C+Y)}{dC}=1$$

In addition, the entropy of a transformed random variable X is Stone (2015, p. 119):

(12) $$H(X)=H(C)+E[log|\frac{dX}{dC}|]$$

Substituting Equation (11) in Equation (12), we have:

(13) H(X) = H(C) + E[log(1)] = H(C)

which, in turn, implies that

(14) H(X|C) = 0

and therefore,

(15) I(X;C) = H(X) − H(X|C) = H(X)

Considering the fact that *I*(*X*; *C*) ≥ 0, ∀*X, C*, we get

(16) H(X)≥0

which implies that the interval within which X lies i.e., *X* ∈ [*a, b*] must satisfy *b* − *a* ≥ 1 since *b* − *a* < 1 ⇒ *H*(*X*) < 0^2^. This observation along with Theorem 2.1 indicate that

(17) 0≤H(X|Y)≤H(X)

with *H*(*X*|*Y*) = *H*(*X*) if and only if X and Y are independent and *H*(*X*|*Y*) = 0 when *X* = *Y*. Now, if Criterion 2.4 is not valid, it must only be the case that

(18) H(X|Y)<H(X|C)⇒H(X|Y)<0

due to Equation (14)^3^. However, this violates the Equation (17).     □

In addition, SBF attenuation must ensure that attenuated effect is maximally reflective of *Y* than *X*, thereby inducing minimum loss of information content of *X* once SBF attenuation is complete.

**Criterion 2.5**. *Preserved information content of* *X* *in* *X*′ *is maximized if attenuation primarily reduces the effect of SBF. Using Theorem* *2.1, we have:*

(19) H(X|Y)≤H(X)

and

(20) $$H(X^{\prime }|Y)\leq H(X^{\prime })$$

Considering Equations (19, 20) and applying Criterion 2.3 and Criterion 2.4, we get:

(21) $$\begin{array}{l} H(X|X^{\prime })\leq H(X|Y)\leq H(X^{\prime }|Y)\leq H(X^{\prime })\\ \Rightarrow H(X|X^{\prime })\leq H(X^{\prime }) \end{array}$$

Criterion 2.5, in turn, indicates that an SBF algorithm that significantly (i.e., in comparison with other such algorithms) maximizes the inequality *H*(*X*′) − *H*(*X*|*X*′) ≥ 0 also attains the maximum frontal activity's information preservation in the resulting fNIRS time series.

### 2.2. Simulation-Based Verification

We simulated (**Figure 2**) four random time series of length 3,000 out of which 600 and 2,500 data points were used as resting and task periods, respectively. We considered four time series to adhere with the number of channels in adapted fNIRS device in this study. In addition, ICA- and PCA-based algorithm require more than a single sequence for their components' calculation. The reason behind 600 and 2,500 data points split was due to the requirement of resting data by PCA-based SBF attenuation algorithm (Zhang et al., 2005) to calculate the global trend in given time series (e.g., global hemodynamic responses such as SBF) in the form of principal components. We chose 600 data points to mimic the 1-min-long resting period in our realtime experimental settings (i.e., 60 s × 10.0 Hz sampling rate of our fNIRS device). This, in turn, resulted in the use of 2,500 data points during the PCA- and ICA-based algorithms analyses. We repeated this random time series simulation for fifty rounds (*M* = 5.76, *SD* = 18.61). Furthermore, we simulated SBF as a Gaussian noise with the same length i.e., 3,000 data points (50 different series one for each round of simulated time series above) with their mean and standard deviation equals 3 times the corresponding simulated channels time series mean and standard deviation (*M* = 15.56, *SD* = 43.25). This noise was also added to the first 600 data points of each of the simulated sequences (i.e., simulated resting portion for PCA-based SBF attenuation) to mimic the global effect of SBF. We report the averaged analysis results of these fifty simulation rounds for simulation-based verification of Criterion 2.1 through Criterion 2.5.

### 2.3. Participants

We conducted three experiments, namely, verbal fluency task (referred to as VFT hereafter), conversation task experiment (referred to as CTE hereafter), and logical memory test (LMT hereafter). Two different groups of 20 (10 females and 10 males, *M* = 70.20, *SD* = 3.78) and 18 (6 females and 12 males, *M* = 72.31, *SD* = 4.16) older adults participated in VFT and CTE. On the other hand, LMT included thirty two (sixteen females and sixteen males, *M* = 20.50, *SD* = 1.80) younger adults. We were unable to record fNIRS data from two participants during VFT and six participants in LMT. Therefore, these individuals were excluded from our analyses.

Choice of VFT was to ensure the correctness as well as effectiveness of our criteria in quantification of the SBF attenuation and frontal activity's information preservation in a fine-grained working memory task. On the other hand, CTE allowed us to evaluate our analysis results in a naturalistic setting. Last, we included LMT to verify our results are unaffected by age of the participants.

All participants were right-handed (confirmed using FLANDERS (Nicholls et al., 2013) handedness questionnaire), were free of neurological and psychiatric disorders, and had no history of hearing impairment. Prior to the data collection, we received approval (approval code: 16-601-1) from the ethical committee at the Advanced Telecommunications Research Institute International (ATR), Kyoto, Japan. All subjects gave written informed consent. Subjects were seated on an easy armchair in the sound-attenuated experimental room, with instructions to fully relax and their eyes closed while resting.

#### 2.3.1. VFT

We adapted the protocol by Takahashi et al. (2011) in their study of SBF effect on fNIRS time series. It consisted of two blocks. Each block consisted of a 6-s-long word generation. The word generation period was preceded with 30 s of silence period and followed by 70 s of control periods. During the word generation period, participants had to generate as many words as possible that started with the syllable that was auditorily presented every 20 s. In the control period, participants were instructed to repeat five syllables: /a/, /i/. /u/, /e/, and /o/. This task was also used by Sato et al. (2016) in their comparative fMRI-fNIRS analysis of reduction of SBF interference on fNIRS time series of frontal brain activity using their proposed GLM-based approach.

We started each experiment by acquiring a 1-min-long resting data, followed by its corresponding VFT session. We provided our participants with a 1-min-long resting break (while staying at their seat with their eyes closed) prior to the commencement of the experiment. We kept the content of the two blocks intact. However, we randomized the order of their occurrence among the participants. We used a speaker as a medium and generated the sequences of VFT using PsychoPy.

#### 2.3.2. CTE

It included a 20-min-long conversation with participants about a site-seeing visit to Yakushima Island in southern Japan (in Japanese). We started by acquiring a 1-min-long resting data, followed by its corresponding 20-min-long experimental session. We provided our participants with a 1-min-long resting break (while staying at their seat with their eyes closed) prior to the commencement of the experiment. We kept the content of conversation intact. A female assistant, who was not aware of the purpose of this study, conversed with our participants. We controlled the conversational session in such a way that the operator was in charge of leading the topic, thereby requiring all the participants to respond to same series of statements and questions [e.g., their most interesting visited site(s), preferred cousin, etc.].

#### 2.3.3. LMT

We adapted the protocol by Basso Moro et al. (2013). It started with participants listening to a 20-s-long story of the LMT (D. Wechsler, 1997) which was immediately followed by participants repeating the narrated story aloud, trying to recall as much of a detail as possible. The recall period lasted for 30 s. We started each experiment by acquiring a 1-min-long resting data, followed by its corresponding LMT session. We provided our participants with a 1-min-long resting break (while staying at their seat with their eyes closed) prior to the commencement of the experiment. We kept the content of the LMT story intact. A female assistant, who was not aware of the purpose of this study, read the stories to the participants through a speaker medium.

### 2.4. Data Acquisition

**Figure 1:**
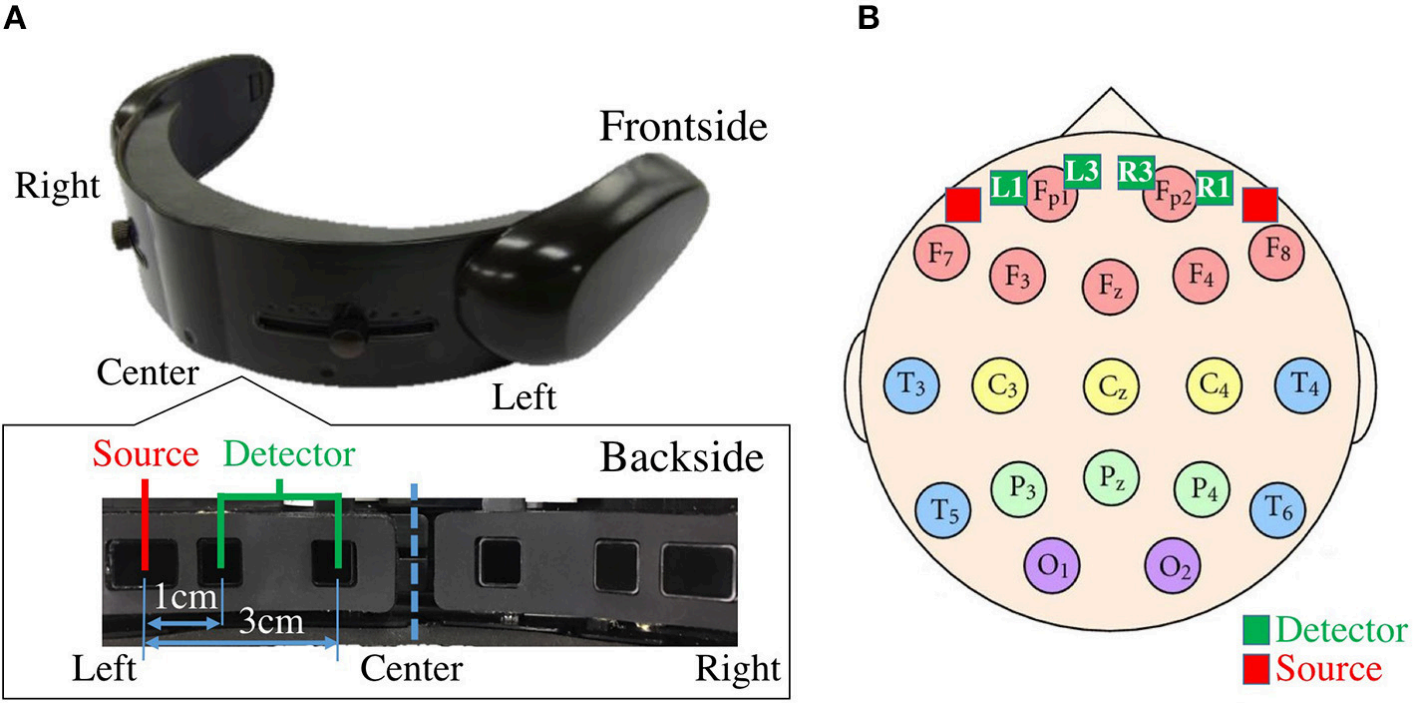
**(A)** fNIRS device in present study. Bottom subplot on left shows arrangement of source-detector of four channels of this device. Distances between short (i.e., 1.0 cm) and long (i.e., 3.0 cm) source and detector of left and right channels are shown. **(B)** Arrangement of 10–20 International Standard System: In this figure, relative locations of channels of fNIRS device in our study (i.e., L1, L3, R1, and R3) are depicted in red (i.e., sources) and green (i.e., detectors) squares. L1, R1, L3, and R3 are channels with short (i.e., 1.0 cm) and long (i.e., 3.0 cm) source-detector distances.

We used functional near infrared spectroscopy (fNIRS) (Ferrari and Quaresima, 2012; Dix et al., 2013) to collect frontal brain activity of the participants. We acquired fNIRS time series data of the participants using a wearable optical topography system “HOT-1000,” developed by Hitachi High-Technologies Corporation (please refer to Figure 1). Participants wore this device on their forehead. It records the frontal brain activity through detection of total blood flow via emitting a wavelength laser light (810 nm) at 10.0 Hz sampling rate. Data acquisition is carried out through four channels (i.e., *Left*_1_, *Left*_3_, *Right*_1_, and *Right*_3_, as shown in Figure 1). Subscripted numerical values that are assigned to these channels specify their respective source-detector distances. In other words, *Left*_1_ and *Right*_1_ have a 1.0 cm and *Left*_3_ and *Right*_3_ have 3.0 cm source-detector distances, respectively. Research findings indicate that short source-detector channels [e.g., 0.5 cm (Takahashi et al., 2011), 1.0 cm (Gagnon et al., 2011), 1.5 cm (Sato et al., 2013, 2016; Kiguchi and Funane, 2014), and 2.0 cm (Yamada et al., 2009)] are mostly representatives of scalp hemodynamics than cortical blood flow (CBF). Moreover, choice of 3.0 cm source-detector distance is customary in NIRS-based studies of brain activity (Yamada et al., 2009; Gagnon et al., 2011; Takahashi et al., 2011; Sato et al., 2013, 2016; Kiguchi and Funane, 2014). It is also worth noting that Zhang et al. (2005) and Kohno et al. (2007) adapted this source-detector distance in their original articles on PCA- and ICA-based SBF attenuation algorithms. Therefore, we primarily report our analysis results on long source-detector channels [*Left*_3_ in the main body of this article and *Right*_3_ in Supplementary Material (SM)].

We placed a laser Doppler tissue blood flow meter probe (FLO-C1, Omegawave Incorporated, Tokyo, Japan) on participants' forehead close to the *Left*_1_ for SBF recording. It collected data from the scalp layer within 1.0 mm from the probe and its analog output was recorded simultaneously alongside the fNIRS recording. This device uses a laser beam with a wavelength of 780.0 nm and has a sampling rate of 10.0 Hz that matches our fNIRS device sampling rate. This device has also been adapted by Takahashi et al. (2011) during their SBF effect study.

To ensure synchronized data acquisition across sensors, we collected data streams for NIRS and SBF through “*labstreaminglayer*” system.

### 2.5. Data Preprocessing

First, we baseline-normalized the data via subtracting the mean of 1-min-long resting period. This step that is customary in fMRI/fNIRS research is based on the assumption that it removes the brain activity that was present prior to the start of the task (as reflected in the time series data recorded during the resting period) and hence does not reflect the effect of the brain activation during the task period. Next and in oder to attenuate the effect of systemic physiological artifacts (Tak and Ye, 2014) (e.g., cardiac pulsations, respiration, etc.) we applied a one-degree polynomial butter worth filter with 0.01 Hz and 0.6 Hz for low and high bandpass. This was followed by linear detrending. Detrending of the signal that is adapted from signal processing and time series analysis and forecasting is a necessary step to ensure that assumptions of stationarity and homoscedasticity (as reflected in wide spread application of linear models in analysis of fNIRS/fMRI time series) are not strongly violated (e.g., due to seasonality and/or repetitive increasing/decreasing patterns) by acquired fNIRS signal.

We adapted the SBF attenuation algorithms by Zhang et al. (2005) (referred to as PCA-based hereafter) and Kohno et al. (2007) (referred to as ICA-based hereafter) in present study. The first approach utilizes an eigenvector-based spatial filtering method that is applied to the rest (baseline) period, thereby removing the first *r* spatial eigenvectors calculated from the baseline data by PCA from the fNIRS time series that are recorded during the task period. We used the resting period time series of the four channels of our device (Figure 1), per participant, for this purpose. Given the combination of short (i.e., *Left*_1_ and *Right*_1_) and long (i.e., *Left*_3_, and *Right*_3_) source-detector channels of our device, the PCA-based algorithm is able to capture the components that best represent global hemodynamics, as noted by Zhang et al. (2005). This is due to the results that indicate the short source-detector channels [e.g., 0.5 cm (Takahashi et al., 2011), 1.0 cm (Gagnon et al., 2011), 1.5 cm (Sato et al., 2013, 2016; Kiguchi and Funane, 2014), and 2.0 cm (Yamada et al., 2009)] are mostly representatives of scalp hemodynamics and the long source-detector channels are suitable for recording of the CBF (Yamada et al., 2009; Gagnon et al., 2011; Takahashi et al., 2011; Sato et al., 2013, 2016; Kiguchi and Funane, 2014). It is worth noting that Zhang et al. (2005) also adapted the 3.0 cm source-detector distance (i.e., similar to *Left*_3_, and *Right*_3_ in our case) for capturing the CBF in their original article on PCA-based SBF attenuation algorithm. On the other hand, ICA-based SBF attenuation algorithm (Kohno et al., 2007) removes the component that has the highest coefficient of spatial uniformity (i.e., the absolute value of the coefficient of variation; Everitt, 1998) among the independent components as a representative of the global scalp-hemodynamic component. Similar to PCA-based algorithm, we used the four channels of our device to determine the component with coefficient of spatial uniformity. It is worth noting that Kohno et al. (2007) also adapted the 3.0 cm source-detector distance (i.e., similar to *Left*_3_, and *Right*_3_ in our case) for capturing the CBF in their original article on ICA-based SBF attenuation algorithm.

Sato et al. (2016) used the PCA-based algorithm after preprocessing steps for SBF attenuation. On the other hand, they utilized the ICA-based algorithm prior to preprocessing steps. These orders for application of SBF attenuation [i.e., preprocessing the fNIRS time series after (in case of ICA-based) and before (in case of PCA-base) SBF attenuation] were reported by Kohno et al. (2007) and Zhang et al. (2005) as well. Therefore, we followed the same orderings while applying these algorithms for attenuation of the effect of SBF on fNIRS time series data of cortical activity of our participants.

Although Zhang et al. (2005) did not provide any specific reason for selection of the number of components, they considered the combination of the first three components (referred to as PC123 hereafter) to be an adequate choice for attenuation of the SBF effect. On the other hand, Sato et al. (2016) argued that the difference between the first three components in contrast with only the first component (referred to as PC1 hereafter) is non-significant. Subsequently, they adapted the first component in their analysis. In present study, we used these two settings. However, considering the explanatory power of the first three components in PCA-based approach (Zhang et al., 2005; Sato et al., 2016), we also included the combination of first two components (referred to as PC12 hereafter) in our analyses. On the other hand, we followed Kohno et al. (2007) and removed the component with highest coefficient of spatial uniformity (referred to as IC1 hereafter) in case of ICA-based SBF attenuation algorithm.

### 2.6. Statistical Analysis

First, we applied Wilcoxon rank sum (i.e., one-sample) test to determine the effect of SBF on fNIRS (i.e., *TE*(*Y* → *X*)) time series of frontal brain activity of participants (in both, VFT and CTE). This was followed by testing Criterion 2.1 through Criterion 2.5 to determine the utility of PCA- and ICA-based algorithms and their respective adapted components (i.e., PC1, PC12, PC123 in case of PCA-based and IC1 in case of ICA-based SBF attenuations) in reduction of the effect of SBF on fNIRS while preserving information content of frontal brain activity.

In case of Criterion 2.1, we performed Kruskal-Wallis test on combination of TE before [i.e., *TE*(*Y* → *X*)] along side after [i.e., *TE*(*Y* → *X*′)] SBF attenuation by each of PC1, PC12, PC123, and IC1 to determine any significance induced by the choice of these components in reduction of TE. This was followed by *post-hoc* paired Wilcoxon rank sum test.

We followed the same steps in case of Criterion 2.2 and Criterion 2.3, replacing TE before [i.e., *TE*(*Y* → *X*)] and after [i.e., *TE*(*Y* → *X*′)] with the “reduction of SBF-related information in frontal brain activity before [i.e., *H*(*Y*|*X*)] and after [i.e., *H*(*Y*|*X*′)] SBF attenuation,” in case of Criterion 2.2, and “reduction of correspondence between SBF and fNIRS time series of frontal brain activity before [i.e., *H*(*X*|*Y*)] and after [i.e., *H*(*X*′|*Y*)] SBF attenuation,” in case of Criterion 2.3, respectively.

In case of Criterion 2.4, we performed Kruskal-Wallis test on combination of *H*(*X*|*Y*) alongside the measured *H*(*X*|*X*′) by each of PC1, PC12, PC123, and IC1 to determine any significance induced by the choice of these components in retaining the correspondence between time series of frontal activity before and after application of SBF attenuation. This was followed by *post-hoc* paired Wilcoxon rank sum test.

In case of Criterion 2.5, we first applied a paired Wilcoxon rank sum (i.e., two-sample) between *H*(*X*|*X*′) and *H*(*X*′) for each of the components (i.e., PC1, PC12, PC123, and IC1) separately, thereby signifying their respective effect in preservation of information content of frontal brain activity. Next, we performed Kruskal-Wallis test on combination of preserved frontal brain activity [i.e., *H*(*X*′) − *H*(*X*|*X*′)] by each of PC1, PC12, PC123, and IC1 to quantitatively determine any significance induced by the choice of these components in such an information preservation. This was followed by *post-hoc* paired Wilcoxon rank sum between every pair of these components, thereby determining the component(s) that significantly maximize such a preservation of information content of frontal brain activity in comparison with other components.

For Kruskal-Wallis, we reported the effect size $r=\sqrt{\frac{\chi^{2}}{N}}$, with *N* denoting the sample size, as suggested by Rosenthal and DiMatteo (2001). In case of Wilcoxon test, we used $r=\frac{W}{\sqrt{N}}$ (Tomczak and Tomczak, 2014) as effect size with *W* denoting the Wilcoxon statistics and *N* is the sample size. All results reported are Bonferroni corrected (i.e., multiplying the *p*-values with the sample size, given the use of non-parametric tests). We used JIDT (Lizier, 2014) for calculation of TE, MI, and conditional entropies. All statistical analyses were carried out in Matlab R2016a environment. We used Gramm (Morel, 2018) for data visualization. We used Python 2.7 for simulated data generation, realtime data acquisition and processing, and information-theoretic measures computation. All statistical analyses were carried out in Matlab R2016a.

## 3. Results

### 3.1. Simulation-Based Verification

**Figure 2:**
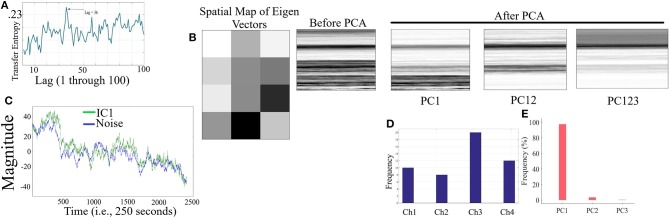
Simulated Time Series **(A)** Grand-average (i.e., fifty rounds of simulation) of computed TE values for Lag = 1, …, 100 (i.e., up to 10 s of lag, considering the sampling rate of 10.0 Hz in our device during real-time data acquisition). Maximum TE was, on average, at Lag = 36 (equivalent to time = 3.6 s of adapted fNIRS device in present study (i.e., sampling rate = 10.0 Hz) **(B)** Sample spatial map of eigenvectors by PCA-based SBF attenuation algorithm along with the sample original simulated time series (in matrix forms for better visualization purpose) before and after application of PC1, PC12, and PC123 components. Distinctive effect of these components on the simulated data is evident in this subplot. All matrices are scaled within [0, 1] interval for ease of comparison. **(C)** Sample Gaussian noise (blue) and sample IC1 component (green) computed by ICA-based SBF attenuation algorithm. In this subplot, the plotted IC1 component pertains to the case in which simulated channel 1 was selected as component with highest coefficient of spatial uniformity. **(D)** Frequency of selected simulated channels with highest coefficient of spatial uniformity by ICA-based SBF attenuation algorithm **(E)** Percentages of the variance-explained by each of the principal components (i.e., PC1, PC12, PC123) of PCA-based SBF attenuation algorithm. Subplots **(D,E)** correspond to all fifty rounds of simulations.

Figure 2A corresponds to the grand-average of the computed TE values for lags 1 through 100 (i.e., up to 10 s of lag, considering the sampling rate of 10.0 Hz of our device during the real-time data acquisition). This subplot indicates that maximum transferred information from simulated noise to simulated channels was, on average, at lag = 36 (i.e., 3.6 s in adapted fNIRS device in present study). This was the lag we used during our analyses. Figure 2B visualizes a sample spatial map of eigenvectors by PCA-based SBF attenuation algorithm along with the sample original simulated time series (in matrix forms for better visualization purpose) before and after application of PC1, PC12, and PC123 components. Distinctive effect of these components on the simulated data is evident in this subplot. We observed significant differences between data before and after application of PC1 [*p* < 0.001, *W*_(7, 998)_ = 24.21, *r* = 0.27, *M*_*Before*_ = 8.46, *SD*_*Before*_ = 12.91, *M*_*PC*1_ = 1.77, *SD*_*PC*1_ = 10.10], PC12 [*p* < 0.001, *W*_(7, 998)_ = 29.74, *r* = 0.33, *M*_*PC*12_ = 0.52, *SD*_*PC*12_ = 10.06], and PC123 [*p* < 0.001, *W*_(7, 998)_ = 35.55, *r* = 0.40, *M*_*PC*123_ = 0.14, *SD*_*PC*123_ = 6.22].

Figure 2C shows a sample Gaussian noise (blue) along with the computed IC1 by ICA-based SBF attenuation algorithm (green) (pertinent to the case in which simulated channel 1 was selected as component with highest coefficient of spatial uniformity). Although this algorithm resulted in selection of different simulated channels as the component with highest coefficient of spatial uniformity (i.e., the absolute value of the coefficient of variation; Everitt, 1998) (Figure 2D, Ch1 = 20.00%, Ch2 = 16.00%, Ch3 = 40.00%, Ch4 = 24.00%), these components were significantly correlated with simulated noise (Ch1: *r* = 0.73, *p* < 0.001, Ch2: *r* = 0.78, *p* < 0.001, Ch3: *r* = 0.78, *p* < 0.001, and Ch4: *r* = 0.89, *p* < 0.001). On the other hand, PCA-based algorithm (Figure 2E) exhibited a higher specificity in selecting the principal components (PC1 = 89.00%, PC12 = 8.10%, PC123 = 2.90%).

#### 3.1.1. Criterion 2.1: *TE*(*Y* → *X*′) ≤ *TE*(*Y* → *X*)

**Figure 3:**
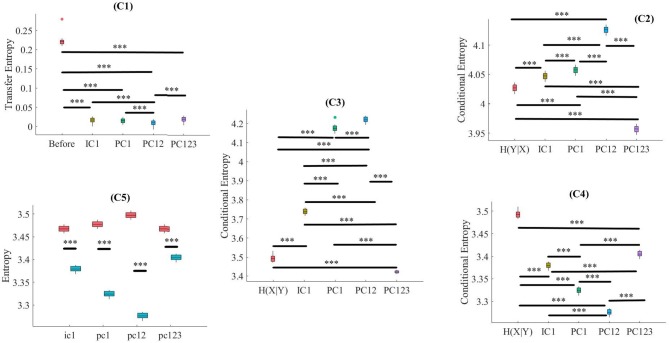
Simulated Data. C1: Criterion 2.1: *TE*(*Y* → *X*′) ≤ *TE*(*Y* → *X*), C2: Criterion 2.2: *H*(*Y*|*X*) ≤ *H*(*Y*|*X*′), C3: Criterion 2.3: *H*(*X*|*Y*) ≤ *H*(*X*′|*Y*), C4: Criterion 2.4: *H*(*X*|*X*′) ≤ *H*(*X*|*Y*), C5: Criterion 2.5: *H*(*X*|*X*′) ≤ *H*(*X*′). In these subplots, IC1, PC1, PC12, and PC123 refer to the measured quantity by these components after their respective SBF attenuation algorithms. Asterisks mark the components with significant difference (^*^p < 0.05, ^**^p < 0.01, ^***^p < 0.001).

Kruskal-Wallis test implied significant effect of choice of components [*p* < 0.001, *H*_(4, 249)_ = 152.35, *r* = 0.61, *M* = 0.22, *SD* = 0.01]. Moreover, *post-hoc* comparison implied that all components significantly reduced SBF effect in comparison with *TE*(*Y* → *X*) [PC1: *p* < 0.001, *W*_(98)_ = 8.61, *r* = 0.86, *M* = 0.02, *SD* = 0.01, PC12: *p* < 0.001, *W*_(98)_ = 8.61, *r* = 0.86, *M* = 0.01, *SD* = 0.01, PC123: *p* < 0.001, *W*_(98)_ = 8.61, *r* = 0.86, *M* = 0.02, *SD* = 0.01, IC1: *p* < 0.001, *W*_(98)_ = 8.61, *r* = 0.86, *M* = 0.02, *SD* = 0.01]. *Post-hoc* comparison indicated that PC12 significantly performed better in attenuation of the effect of SBF than PC1 [*p* < 0.001, *W*_(98)_ = 4.11, *r* = 0.41], PC123 [*p* < 0.001, *W*_(98)_ = 6.34, *r* = 0.63], and IC1 [*p* < 0.001, *W*_(98)_ = 4.90, *r* = 0.49]. On the other hand, we observed non-significant differences between PC1 and PC123 [*p* = 0.14, *W*_(98)_ = 1.52, *r* = 0.15], PC1 and IC1 [*p* = 0.12, *W*_(98)_ = 1.52, *r* = 0.15], as well as PC123 and IC1 [*p* = 0.13, *W*_(98)_ = 1.52, *r* = 0.15]. Figure 3, subplot C1, illustrates these results.

#### 3.1.2. Criterion 2.2: *H*(*Y*|*X*) ≤ *H*(*Y*|*X*′)

Kruskal-Wallis implied a significant effect of choice of components [*p* < 0.001, *H*_(4, 249)_ = 234.13, *r* = 0.94]. *Post-hoc* comparison indicated a significant reduction of SBF-related information in frontal brain activity of participants by PC1 [*p* < 0.001, *W*_(98)_ = 8.61, *r* = 0.86, *M*_*H*(*Y*|*X*)_ = 4.03, *SD*_*H*(*Y*|*X*)_ = 0.01, *M*_*PC*1_ = 4.06, *SD*_*PC*1_= 0.03], PC12 [*p* < 0.001, *W*_(98)_ = 8.61, *r* = 0.86, *M*_*PC*12_ = 4.13, *SD*_*PC*12_ = 0.01]. Interestingly, application of PC123 resulted in a significantly reduced information [*p* < 0.001, *W*_(98)_ = 8.6, *r* = 0.86, *M*_*PC*12_ = 3.96, *SD*_*PC*12_ = 0.02], and IC1 [*p* < 0.001, *W*_(98)_ = 8.61, *r* = 0.86, *M*_*IC*1_ = 4.05, *SD*_*IC*1_ = 0.02]. Additionally, this comparison indicated that adaptation of PC1 resulted in a significantly more reduced SBF-related information than PC123 [*p* < 0.001, *W*_(98)_ = 8.61, *r* = 0.86] as well as IC1 [*p* < 0.001, *W*_(98)_ = 6.61, *r* = 0.86]. In addition, we found PC12 significantly more effective than PC1 [*p* < 0.001, *W*_(98)_ = 8.61, *r* = 0.86], PC123 [*p* < 0.001, *W*_(98)_ = 8.61, *r* = 0.86] and IC1 [*p* < 0.001, *W*_(98)_ = 8.61, *r* = 0.86]. Last, we observed a significant differences between IC1 and PC123 was significant [*W*_(98)_ = 8.61, *r* = 0.86]. Figure 3, subplot C2, shows these results.

#### 3.1.3. Criterion 2.3: *H*(*X*|*Y*) ≤ *H*(*X*′|*Y*)

Kruskal-Wallis indicated a significant effect of choice of components in resulting fNIRS data [*p* < 0.001, *H*_(4, 249)_ = 238.00, *r* = 0.96]. Whereas *post-hoc* comparison suggested that PC123 was ineffective in satisfying this Criterion 2.3 [PC123: *p* < 0.001, *W*_(98)_ = 8.61, *r* = 0.86, *M*_*H*(*X*|*Y*)_ = 3.49, *SD*_*H*(*X*|*Y*)_ = 0.04, *M*_*PC*123_ = 3.421, *SD*_*PC*123_ = 0.04] all other components were significantly effective in reducing the degree of correspondence between SBF and fNIRS time series of frontal brain activity [PC1: *p* < 0.001, *W*_(98)_ = 8.61, *r* = 0.86, *M*_*PC*1_ = 4.17, *SD*_*PC*1_ = 0.01, PC12: *p* < 0.001, *W*_(98)_ = 8.61, *r* = 0.86, *M*_*PC*12_ = 4.22, *SD*_*PC*12_ = 0.01, IC1: *p* < 0.001, *W*_(98)_ = 8.61, *r* = 0.86, *M*_*IC*1_ = 3.74, *SD*_*IC*1_ = 0.03]. In addition, this comparison indicated that adaptation of PC12 yielded a significantly better performance than PC1 [*p* < 0.001, *W*_(98)_ = 8.23, *r* = 0.82], PC123 [*p* < 0.001, *W*_(98)_ = 8.61, *r* = 0.86], as well as IC1 [*p* < 0.001, *W*_(98)_ = 8.61, *r* = 0.86]. Moreover, PC1 was significantly more effective than PC123 [*p* < 0.001, *W*_(98)_ = 8.61, *r* = 0.86] and IC1 [*p* < 0.001, *W*_(98)_ = 8.61, *r* = 0.86]. Additionally, IC1 was significantly different from PC123 [*W*_(98)_ = 8.61, *r* = 0.86]. Figure 3, subplot C3, plots these results.

#### 3.1.4. Criterion 2.4: *H*(*X*|*X*′) ≤ *H*(*X*|*Y*)

Kruskal-Wallis indicated a significant effect of choice of components [*p* < 0.001, *H*_(4, 249)_ = 239.04, *r* = 0.96]. *Post-hoc* comparison suggested that all components were significantly effective in retaining the correspondence between time series of frontal activity before and after application of SBF attenuation [PC1: *p* < 0.001, *W*_(98)_ = 8.61, *r* = 0.86, *M*_*H*(*X*|*Y*)_ = 3.49, *SD*_*H*(*X*|*Y*)_ = 0.04 *M*_*PC*1_ = 3.32, *SD*_*PC*1_ = 0.01, PC12: *p* < 0.001, *W*_(98)_ = 8.61, *r* = 0.86, *M*_*PC*1_ = 3.28, *SD*_*PC*12_ = 0.03, PC123: *p* < 0.001, *W*_(98)_ = 8.61, *r* = 0.86, *M*_*PC*123_ = 3.41, *SD*_*PC*123_ = 0.05, IC1: *p* < 0.001, *W*_(98)_ = 8.61, *r* = 0.86, *M*_*IC*1_ = 3.38, *SD*_*IC*1_ = 0.01]. Additionally, this comparison implied that PC12 resulted in significantly higher correspondence between time series of frontal brain activity before and after SBF attenuation than PC1 [*p* < 0.001, *W*_(98)_ = 8.61, *r* = 0.86], PC123 [*p* < 0.001, *W*_(98)_ = 8.61, *r* = 0.86], as well as IC1 [*p* < 0.001, *W*_(98)_ = 8.613828 *r* = 0.861383]. Similarly, PC1 performed significantly better than PC123 [*p* < 0.001, *W*_(98)_ = 8.61, *r* = 0.86] and IC1 [*p* < 0.001, *W*_(98)_ = 8.61, *r* = 0.86]. Lastly, IC1 was significantly more effective than PC123 [*p* < 0.001, *W*_(98)_ = 8.61, *r* = 0.86]. Figure 3, subplot C4, shows these results.

#### 3.1.5. Criterion 2.5: *H*(*X*|*X*′) ≤ *H*(*X*′)

Pairwise Wilcoxon rank sum suggested that all components preserved information content of frontal activity [PC1:*p* < 0.001, *W*_(98)_ = 8.61, *r* = 0.86, $M_{H\left(X|X^{\prime }\right)}$ = 3.32, ${SD}_{H\left(X|X^{\prime }\right)}$ = 0.05, $M_{H\left(X^{\prime }\right)}$ = 3.48, ${SD}_{H\left(X^{\prime }\right)}$ = 0.06, PC12:*p* < 0.001, *W*_(98)_ = 8.61, *r* = 0.86, $M_{H\left(X^{\prime }\right)}$ = 3.50, ${SD}_{H\left(X^{\prime }\right)}$ = 0.07, PC123: *p* < 0.001, *W*_(98)_ = 8.61, *r* = 0.86, $M_{H\left(X^{\prime }\right)}$ = 3.46, ${SD}_{H\left(X^{\prime }\right)}$ = 0.07, IC1: *p* < 0.001, *W*_(98)_ = 8.61, *r* = 0.86, $M_{H\left(X^{\prime }\right)}$ = 3.48, ${SD}_{H\left(X^{\prime }\right)}$ = 0.06]. Figure 3, subplot C5, depicts these results.

**Figure 4:**
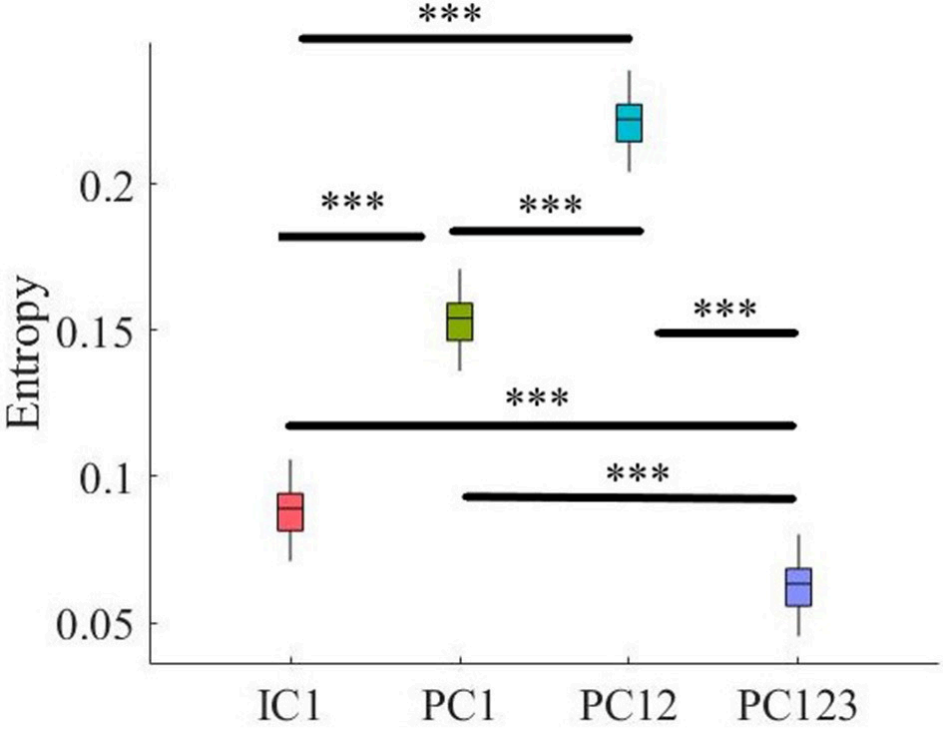
Comparison of maximal preservation of information content of frontal brain activity (i.e., *H*(*X*′) − *H*(*X*|*X*′) ≥ 0, Criterion 2.5) after application of PCA- and ICA-based SBF attenuation algorithms on simulated data. Asterisks mark the components with significant difference (^*^p < 0.05, ^**^p < 0.01, ^***^p < 0.001).

Kruskal-Wallis test indicated a significant effect of choice of component on preservation of information content of fNIRS time series [i.e., *H*(*X*′) − *H*(*X*|*X*′) ≥ 0] [*p* < 0.001, *H*_(3, 199)_ = 186.57, *r* = 0.94]. *Post-hoc* paired comparison implied a significantly higher preservation of information content of frontal brain activity with respect to PC12 in comparison with PC1 [*p* < 0.001, *W*_(98)_ = 8.61, *r* = 0.86, *M*_*PC*1_ = 0.15, *SD*_*PC*1_ = 0.01, *M*_*PC*12_ = 0.22, *SD*_*PC*1_ = 0.01], PC123 [*p* < 0.001, *W*_(98)_ = 8.61, *r* = 0.86, *M*_*PC*123_ = 0.06, *SD*_*PC*1_ = 0.03], as well as IC1 [*p* < 0.001, *W*_(98)_ = 8.61, *r* = 0.86, *M*_*IC*1_ = 0.09, *SD*_*IC*1_ = 0.01]. PC1 was significantly more effective than and PC123 [*p* < 0.001, *W*_(98)_ = 8.61, *r* = 0.86] and IC1 [*p* < 0.001, *W*_(98)_ = 8.61, *r* = 0.86]. Last, we found that IC1 was significantly more effective than PC123 [*p* < 0.001, *W*_(98)_ = 8.61, *r* = 0.86]. Figure 4 shows these results.

### 3.2. VFT

**Figure 5:**
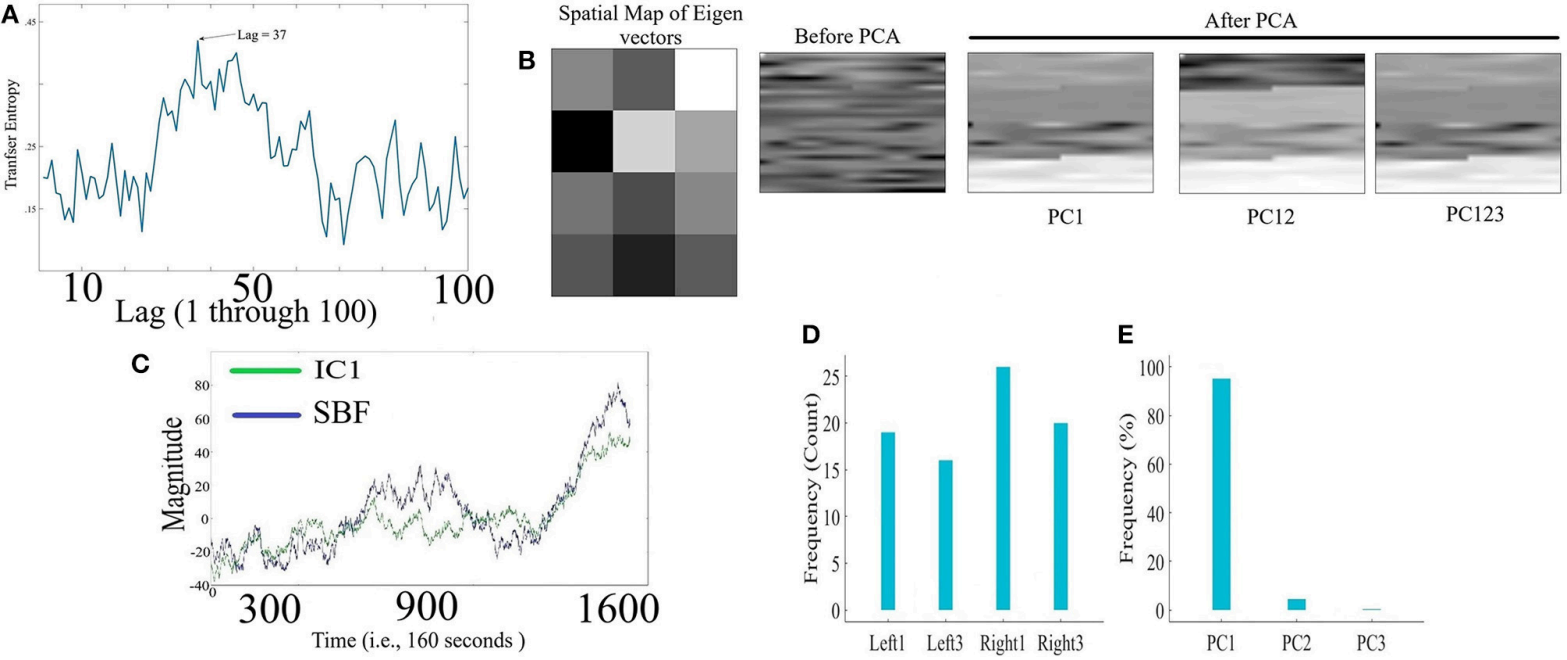
VFT - **(A)** Grand-average of TE values for Lag = 1, …, 100 (i.e., up to 10 s of lag, sampling rate of 10.0 Hz in our device). Maximum TE was at Lag = 37 (equivalent to time = 3.7 s) **(B)** Sample spatial map of eigenvectors pertinent to first three principal components along with a sample PFC time series of a participant (in matrix forms for better visualization purpose) before and after application of PC1, PC12, and PC123 components. Distinctive effect of these components on the simulated data is evident in this subplot. All matrices are scaled within [0, 1] interval for ease of comparison. **(C)** Sample SBF (blue) and the corresponding IC1 component (green) computed by ICA-based SBF attenuation algorithm. The depicted IC1 component pertains to the case in which *Right*_1_ was selected as component with highest coefficient of spatial uniformity. **(D)** Frequency of selected channels as a component with highest coefficient of spatial uniformity by ICA-based SBF attenuation algorithm for VFT dataset. **(E)** Percentages of the variance-explained by each of the principal components (i.e., PC1, PC12, PC123) of PCA-based SBF attenuation algorithm for VFT dataset.

Figure 5A corresponds to the grand-average of the computed TE values for lags 1 through 100 (i.e., up to 10 s of lag, considering the sampling rate of 10.0 Hz of our device) in VFT. This subplot indicates that maximum SBF transferred information was, on average, at lag = 37 (i.e., 3.7 s). This was the lag we used during our analyses. Wilcoxon rank sum test implied significant effect of SBF on fNIRS time series of frontal brain activity of participants while performing VFT working memory task [*p* < 0.001, *W*_(125)_ = 7.82, *M* = 0.44, *SD* = 0.15, *r* = 0.87].

Figure 5B visualizes a sample spatial map of eigenvectors by PCA-based SBF attenuation algorithm along with a sample participant's PFC time series (in matrix forms for better visualization purpose) before and after application of PC1, PC12, and PC123 components. Distinctive effect of these components on participant's PFC time series is evident in this subplot. We observed significant differences between PFC time series before and after application of PC1 [*p* < 0.001, *W*_(2, 398)_ = 6.67, *r* = 0.14, *M*_*Before*_ = 149.50, *SD*_*Before*_ = 19.13, *M*_*PC*1_ = 144.50, *SD*_*PC*1_ = 18.76], PC12 [*p* < 0.001, *W*_(2, 398)_ = 12.98, *r* = 0.27, *M*_*PC*12_ = 139.27, *SD*_*PC*12_ = 8.76], and PC123 [*p* < 0.001, *W*_(2, 398)_ = 6.66, *r* = 0.14, *M*_*PC*123_ = 145.00, *SD*_*PC*123_ = 16.23].

Figure 5C shows a sample SBF (blue) along with the computed IC1 by ICA-based SBF attenuation algorithm (green) (pertinent to the case in which *Right*_1_ was selected as the component with highest coefficient of spatial uniformity). Although this algorithm resulted in selection of different channels as the component with highest coefficient of spatial uniformity (Figure 5D, *Left*_1_ = 23.46%, *Left*_3_ = 19.75%, *Right*_1_ = 32.10%, *Right*_3_ = 24.69%), these components were significantly correlated with SBF (*Left*_1_: 0.74, *p* < 0.001, *Left*_3_: *r* = 0.76, *p* < 0.001, *Right*_1_: *r* = 0.80, *p* < 0.001, and *Right*_3_: *r* = 0.86, *p* < 0.001). On the other hand, PCA-based algorithm (Figure 5E) exhibited a higher specificity in selecting the principal components (PC1 = 95.15%, PC12 = 4.50%, PC123 = 0.35%).

In what follows, we examine the effectiveness of PCA- and ICA-based SBF attenuation algorithms on reduction of observed impact of SBF on fNIRS frontal brain activity as well as their utility in preservation of information content of this activity in resulting fNIRS time series through investigation of Criterion 2.1 through Criterion 2.5.

#### 3.2.1. Criterion 2.1: *TE*(*Y* → *X*′) ≤ *TE*(*Y* → *X*)

**Figure 6:**
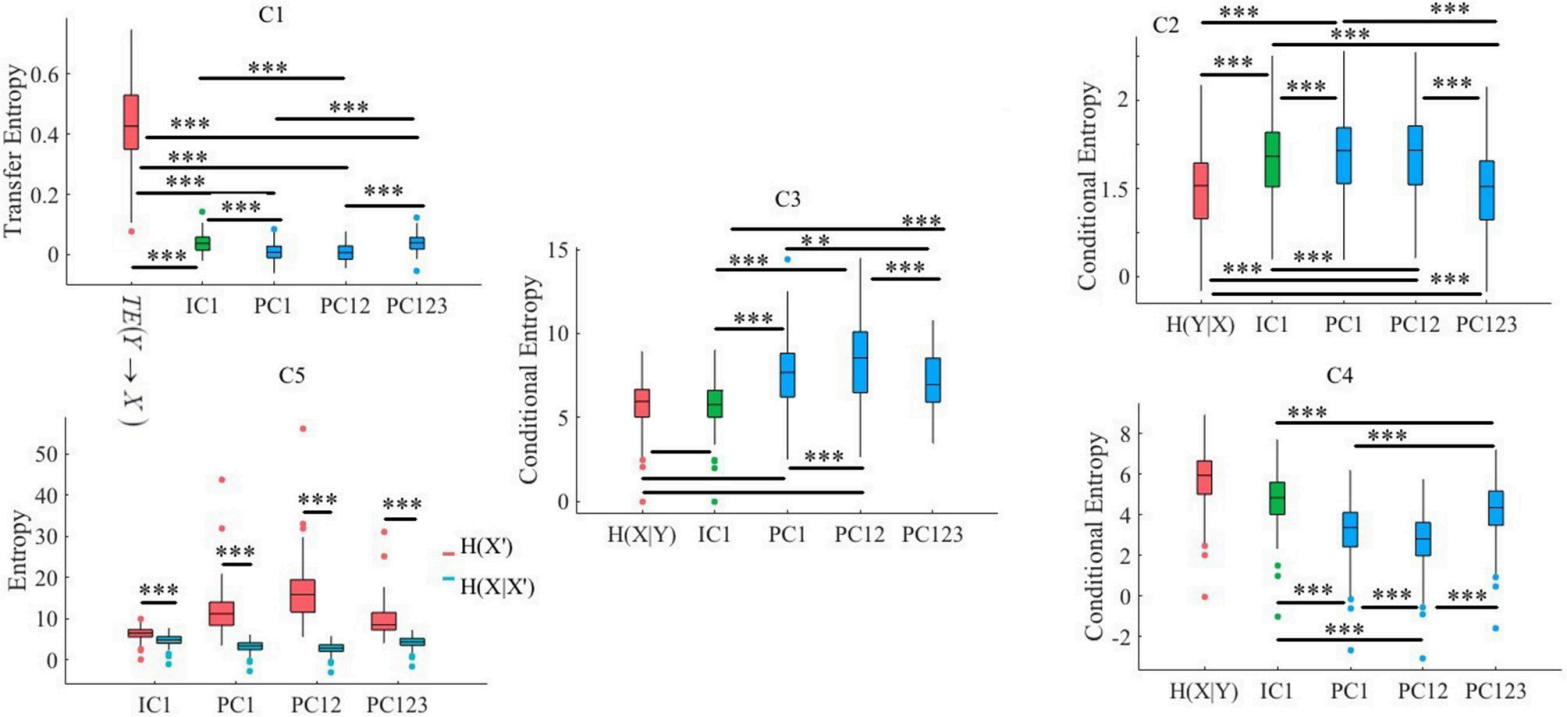
VFT. C1: Criterion 2.1: *TE*(*Y* → *X*′) ≤ *TE*(*Y* → *X*), C2: Criterion 2.2: *H*(*Y*|*X*) ≤ *H*(*Y*|*X*′), C3: Criterion 2.3: *H*(*X*|*Y*) ≤ *H*(*X*′|*Y*), C4: Criterion 2.4: *H*(*X*|*X*′) ≤ *H*(*X*|*Y*), C5: Criterion 2.5: *H*(*X*|*X*′) ≤ *H*(*X*′). In these subplots, IC1, PC1, PC12, and PC123 refer to the measured quantity by these components after their respective SBF attenuation algorithms. Asterisks mark the components with significant difference (*p < 0.05, **p < 0.01, ***p < 0.001).

Kruskal-Wallis test implied significant effect of choice of components [*p* < 0.001, *H*_(4, 629)_ = 237.33, *r* = 0.61]. Moreover, *post-hoc* comparison implied that all components significantly reduced SBF effect in comparison with *TE*(*Y* → *X*) [PC1: *p* < 0.001, *W*_(250)_ = 7.82, *M* = 0.009, *SD* = 0.03, *r* = 0.49, PC12: *p* < 0.001, *W*_(250)_ = 7.82, *M* = 0.009, *SD* = 0.03, *r* = 0.49, PC123: *p* < 0.001, *W*_(250)_ = 7.82, *M* = 0.04, *SD* = 0.03, *r* = 0.49, IC1: *p* < 0.001, W_(250)_ = 7.82, *M* = 0.04, *SD* = 0.03, *r* = 0.49]. On the other hand, whereas this comparison indicated non-significant difference between PC1 and PC12 [*p* = 1.00, *W*_(250)_ = 0.002, *r* = 0.0] as well as PC123 and IC1 [*p* = 0.41, *W*_(250)_ = 0.83, *r* = 0.05), we found significant differences in reduction of TE between PC1 and PC123 [*p* < 0.001, *W*_(250)_ = 6.38, *r* = 0.40], PC1 and IC1[*p* < 0.001, *W*_(250)_ = 6.90, *r* = 0.43], PC12 and PC123 [*p* = < 0.001, *W*_(250)_ = 6.62, *r* = 0.42], as well as PC12 and IC1 [*p* < 0.001, *W*_(250)_ = 6.51, *r* = 0.41]. Figure 6, subplot C1, illustrates these results.

#### 3.2.2. Criterion 2.2: *H*(*Y*|*X*) ≤ *H*(*Y*|*X*′)

Kruskal-Wallis implied a significant effect of choice of components [*p* < 0.001, *H*_(4, 629)_ = 47.37, *r* = 0.27]. *Post-hoc* comparison indicated a significant reduction of SBF-related information in frontal brain activity of participants by PC1 [*p* < 0.001, *W*_(250)_ = 7.82, *M*_*H*(*Y*|*X*)_ = 1.52, *SD*_*H*(*Y*|*X*)_ = 0.50, *M*_*PC*1_ = 1.62, *SD*_*PC*1_ = 0.50, *r* = 0.49], PC12 [*p* < 0.001, *W*_(250)_ = 7.81, *M*_*PC*12_ = 1.63, *SD*_*PC*12_ = 0.50, *r* = 0.49], and IC1 [*p* < 0.001, *W*_(250)_ = 7.81, *M*_*IC*1_ = 1.57, *SD*_*IC*1_ = 0.50, *r* = 0.49]. However, it was non-significant with respect to PC123 [*p* = 0.20, *W*_(250)_ = 1.28, *M*_*PC*123_ = 1.53, *SD*_*PC*1_ = 0.50, *r* = 0.08]. Additionally, this comparison indicated that adaptation of PC1 resulted in a significantly more reduced SBF-related information than PC123 [*p* < 0.001, *W*_(250)_ = 7.81, *r* = 0.49] as well as IC1 [*p* < 0.001, *W*_(250)_ = 7.79, *r* = 0.49]. Similarly, we found PC12 significantly more effective than PC123 [*p* < 0.001, *W*_(250)_ = 7.82, *r* = 0.49] and IC1 [*p* < 0.001, *W*_(250)_ = 7.55, *r* = 0.49]. Additionally, difference between IC1 and PC123 was significant [*p* < 0.001, *W*_(250)_ = 7.82, *r* = 0.49]. However, we found non-significant difference between PC1 and PC12 [*p* = 0.15, *W*_(250)_ = 1.46, *r* = 0.09]. Figure 6, subplot C2, shows these results.

#### 3.2.3. Criterion 2.3: *H*(*X*|*Y*) ≤ *H*(*X*′|*Y*)

Kruskal-Wallis indicated a significant effect of choice of components in resulting fNIRS data [*p* < 0.001, *H*_(4, 629)_ = 83.87, *r* = 0.36]. *Post-hoc* comparison suggested that all components were significantly effective in reducing the degree of correspondence between SBF and fNIRS time series of frontal brain activity [PC1:*p* < 0.001, *W*_(250)_ = 7.62, *M*_*H*(*X*|*Y*)_ = 5.94, *SD*_*H*(*X*|*Y*)_ = 1.67, *M*_*PC*1_= 7.66, *SD*_*PC*1_ = 2.04, *r* = 0.48, PC12: *p* < 0.001, *W*_(250)_ = 7.82, *M*_*PC*12_ = 8.37, *SD*_*PC*12_ = 2.26, *r* = 0.49, PC123: *p* < 0.001, *W*_(250)_ = 7.82, *M*_*PC*123_ = 7.16, *SD*_*PC*123_ = 1.71, *r* = 0.49, IC1: *p* < 0.001, *W*_(250)_ = 3.90, *M*_*IC*1_ = 5.81, *SD*_*IC*1_ = 1.64, *r* = 0.25]. In addition, this comparison indicated that adaptation of PC12 yielded a significantly better performance than PC1 [*p* < 0.001, *W*_(250)_ = 5.10, *r* = 0.32], PC123 [*p* < 0.001, *W*_(250)_ = 5.21, *r* = 0.33], as well as IC1 [*p* < 0.001, *W*_(250)_ = 7.79, *r* = 0.49]. Moreover, PC1 was significantly more effective than PC123 [*p* < 0.01, *W*_(250)_ = 2.92, *r* = 0.18] and IC1 [*p* < 0.001, *W*_(250)_ = 7.53, *r* = 0.47]. Additionally, PC123 was significantly different from IC1 [*p* < 0.001, *W*_(250)_ = 7.65, *r* = 0.48]. Figure 6, subplot C3, plots these results.

#### 3.2.4. Criterion 2.4: *H*(*X*|*X*′) ≤ *H*(*X*|*Y*)

Kruskal-Wallis indicated a significant effect of choice of components [*p* < 0.001, *H*_(4, 629)_ = 136.25, *r* = 0.46]. *Post-hoc* comparison suggested that all components were significantly effective in retaining the correspondence between time series of frontal activity before and after application of SBF attenuation [PC1: *p* < 0.001, *W*_(250)_ = 7.25, *M*_*H*(*X*|*Y*)_ = 5.94, *SD*_*H*(*X*|*Y*)_ = 1.67, *M*_*PC*1_ = 3.32, *SD*_*PC*1_ = 1.64, *r* = 0.46, PC12: *p* < 0.001, *W*_(250)_ = 7.54, *M*_*PC*12_ = 2.88, *SD*_*PC*12_ = 1.63, *r* = 0.48, PC123:*p* < 0.001, *W*_(250)_ = 5.49, *M*_*PC*123_ = 4.38, *SD*_*PC*123_ = 1.65, *r* = 0.35, IC1: *p* < 0.001, *W*_(250)_ = 4.40, *M*_*IC*1_ = 4.84, *SD*_*IC*1_ = 1.63, *r* = 0.28]. Additionally, this comparison implied that PC12 resulted in significantly higher correspondence between time series of frontal brain activity before and after SBF attenuation than PC1 [*p* < 0.001, *W*_(250)_ = 7.82, *r* = 0.49], PC123 [*p* < 0.001, *W*_(250)_ = 7.82, *r* = 0.49], as well as IC1 [*p* < 0.001, *W*_(250)_ = 7.82, *r* = 0.49]. This was followed by significantly better performance by PC1 in contrast with PC123 [*p* < 0.001, *W*_(250)_ = 7.82, *r* = 0.49] and IC1 [*p* < 0.001, *W*_(250)_ = 7.82, *r* = 0.49]. Lastly, PC123 was significantly more effective than IC1 [*p* = 0.001, *W*_(250)_ = 7.82, *r* = 0.49]. Figure 6, subplot C4, shows these results.

#### 3.2.5. Criterion 2.5: *H*(*X*|*X*′) ≤ *H*(*X*′)

Pairwise Wilcoxon rank sum suggested that all components preserved information content of frontal activity [PC1: *p* < 0.001 *W*_(250)_ = 7.76, $M_{H\left(X|X^{\prime }\right)}$ = 3.32, ${SD}_{H\left(X|X^{\prime }\right)}$ = 1.64, $M_{H\left(X^{\prime }\right)}$ = 11.89, ${SD}_{H\left(X^{\prime }\right)}$ = 5.73, *r* = 0.49, PC12: *p* < 0.001 *W*_(250)_ = 7.81, $M_{H\left(X|X^{\prime }\right)}$ = 2.88, ${SD}_{H\left(X|X^{\prime }\right)}$ = 1.63, $M_{H\left(X^{\prime }\right)}$ = 16.75, ${SD}_{H\left(X^{\prime }\right)}$ = 7.61, *r* = 0.49, PC123: *p* < 0.001 *W*_(250)_ = 7.62, $M_{H\left(X|X^{\prime }\right)}$ = 4.38, ${SD}_{H\left(X|X^{\prime }\right)}$ = 1.65, $M_{H\left(X^{\prime }\right)}$ = 9.66, ${SD}_{H\left(X^{\prime }\right)}$ = 4.23, *r* = 0.48, IC1: *p* < 0.001, *W*_(250)_ = 5.37, $M_{H\left(X|X^{\prime }\right)}$ = 4.84, ${SD}_{H\left(X|X^{\prime }\right)}$ = 1.63, $M_{H\left(X^{\prime }\right)}$ = 6.38, ${SD}_{H\left(X^{\prime }\right)}$ = 1.82, *r* = 0.34]. Figure 6, subplot C5, depicts these results.

**Figure 7:**
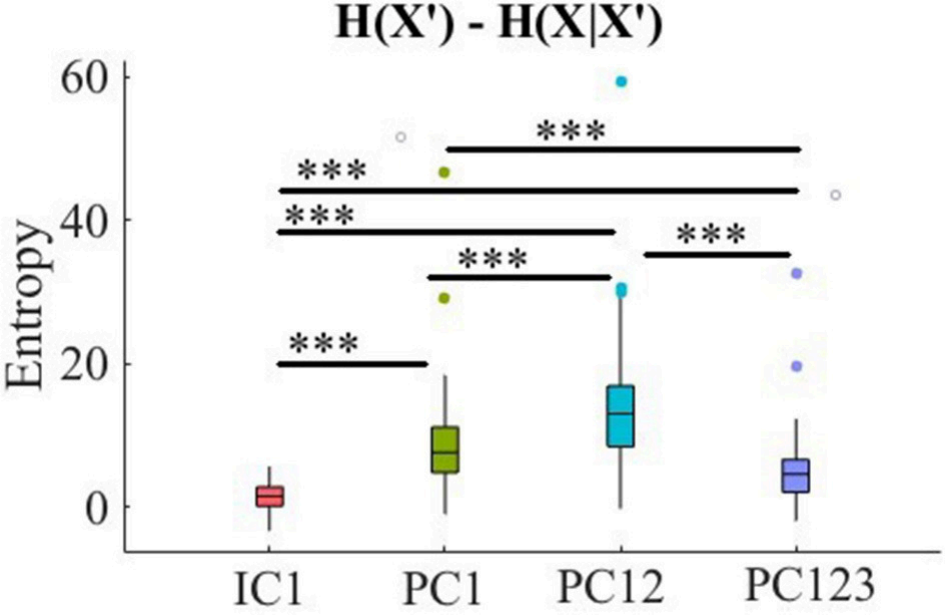
Comparison of maximal preservation of information content of frontal brain activity (i.e., *H*(*X*′) − *H*(*X*|*X*′) ≥ 0, Criterion 2.5) after application of PCA- and ICA-based SBF attenuation algorithms on VFT. Asterisks mark the components with significant difference (^*^p < 0.05, ^**^p < 0.01, ^***^p < 0.001).

Kruskal-Wallis test indicated a significant effect of choice of component on preservation of information content of fNIRS time series [i.e., *H*(*X*′) − *H*(*X*|*X*′) ≥ 0] [*p* < 0.001, *H*_(3, 503)_ = 68.52, *r* = 0.37]. *Post-hoc* paired comparison implied a significantly higher preservation of information content of frontal brain activity with respect to PC12 in comparison with PC1 [*p* < 0.001, *W*_(250)_ = 7.82, *M*_*PC*12_ = 13.86, *SD*_*PC*12_ = 8.42, *M*_*PC*1_ = 8.57, *SD*_*PC*1_ = 6.47], PC123 [*p* < 0.001, *r* = 0.49, *W*_(250)_ = 7.52, *M*_*PC*123_ = 5.27, *SD*_*PC*123_ = 4.86, *r* = 0.47], as well as IC1 [*p* < 0.001, *W*_(250)_ = 7.82, *M*_*IC*1_ = 1.54, *SD*_*IC*1_ = 2.15, *r* = 0.49]. Furthermore, PC1 was significantly more effective than PC123 [*p* < 0.001, *W*_(250)_ = 6.32, *r* = 0.40] and IC1 [*p* < 0.001, *W*_(250)_ = 7.82, *r* = 0.49]. Lastly, we found PC123 significantly better than IC1 [*p* < 0.001, *W*_(250)_ = 7.72, *r* = 0.49]. Figure 7 shows these results.

### 3.3. CTE

**Figure 8:**
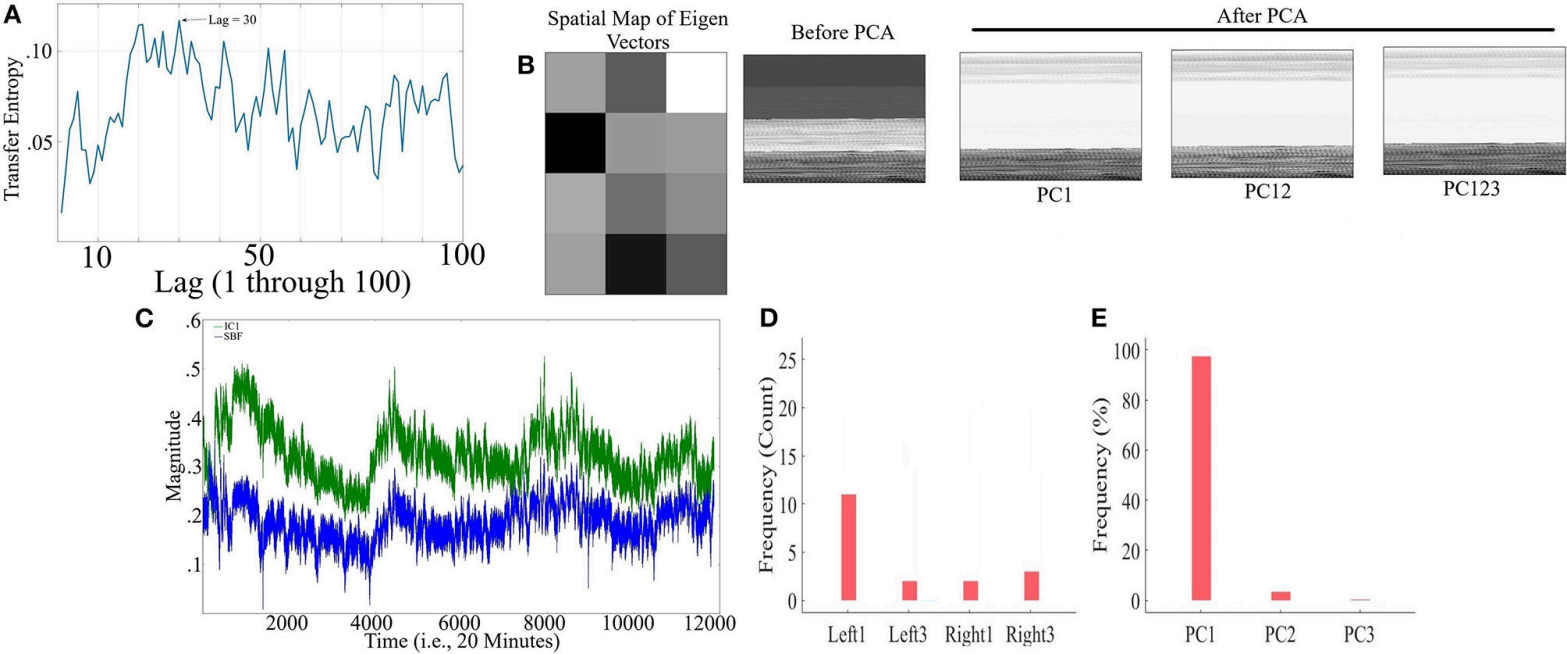
CTE - **(A)** Grand-average of TE values for Lag = 1, …, 100 (i.e., up to 10 s of lag, considering the sampling rate of 10.0 Hz in our device). Maximum TE was at Lag = 30 (equivalent to time = 3.0 s) **(B)** Sample spatial map of eigenvectors by PCA-based SBF attenuation algorithm along with a sample participant's PFC time series (in matrix forms for better visualization purpose) before and after application of PC1, PC12, and PC123 components. Distinctive effect of these components on participant's PFC time series is evident in this subplot. All matrices are scaled within [0, 1] interval for ease of comparison. **(C)** Sample SBF (blue) and the corresponding IC1 component computed by ICA-based SBF attenuation algorithm (blue). The depicted IC1 component pertains to the case in which *Right*_1_ was selected as component with highest coefficient of spatial uniformity. **(D)** Frequency of selected channels as a component with highest coefficient of spatial uniformity by ICA-based SBF attenuation algorithm for CTE dataset. **(E)** Percentages of the variance-explained by each of the principal components (i.e., PC1, PC12, PC123) of PCA-based SBF attenuation algorithm for CTE dataset.

Figure 8A corresponds to the grand-average of the computed TE values for lags 1 through 100 (i.e., up to 10 s of lag, considering the sampling rate of 10.0 Hz) in CTE. This subplot indicates that maximum SBF transferred information was, on average, at lag = 30 (i.e., 3.0 s). This was the lag we used during our analyses. Wilcoxon rank sum test implied significant effect of SBF on fNIRS time series of frontal brain activity of participants during conversation [*p* < 0.001, *W*_(17)_ = 3.72, *M* = 0.13, *SD* = 0.13].

Figure 8B visualizes a sample spatial map of eigenvectors by PCA-based SBF attenuation algorithm along with a sample participant's PFC time series (in matrix forms for better visualization purpose) before and after application of PC1, PC12, and PC123 components. Distinctive effect of these components on participant's PFC time series is evident in this subplot. All matrices are scaled within [0, 1] interval for ease of comparison. We observed significant differences between PFC time series before and after application of PC1 [*p* < 0.001, *W*_(87, 998)_ = 78.24, *r* = 0.26, *M*_*Before*_ = 0.71, *SD*_*Before*_ = 2.02, *M*_*PC*1_ = 0.03, *SD*_*PC*1_ = 0.28], PC12 [*p* < 0.001, *W*_(87, 998)_ = 102.05, *r* = 0.34, *M*_*PC*12_ = 0.004, *SD*_*PC*12_ = 0.13], and PC123 [*p* < 0.001, *W*_(87, 998)_ = 76.69, *r* = 0.26, *M*_*PC*123_ = 0.02, *SD*_*PC*123_ = 0.37].

Figure 8C shows a sample SBF (blue) along with the computed IC1 by ICA-based SBF attenuation algorithm (green) (pertinent to the grand-average of the cases in which *Right*_1_ was selected as the component with highest coefficient of spatial uniformity). Although this algorithm resulted in selection of different channels as the component with highest coefficient of spatial uniformity (Figure 8D, *Left*_1_ = 57.89%, *Left*_3_ = 10.53%, *Right*_1_ = 10.53%, *Right*_3_ = 21.05%), these components were significantly correlated with simulated noise (*Left*_1_: 0.79, *p* < 0.001, *Left*_3_: *r* = 0.67, *p* < 0.001, *Right*_1_: *r* = 0.81, *p* < 0.001, and *Right*_3_: *r* = 0.76, *p* < 0.001). On the other hand, PCA-based algorithm (Figure 8E) exhibited a higher specificity in selecting the principal components (PC1 = 97.42%, PC12 = 2.43%, PC123 = 0.15%).

In what follows, we examine the effectiveness of PCA- and ICA-based SBF attenuation algorithms on reduction of observed impact of SBF on fNIRS frontal brain activity as well as their utility in preservation of information content of this activity in resulting fNIRS time series through investigation of Criterion 2.1 through Criterion 2.5.

#### 3.3.1. Criterion 2.1: *TE*(*Y* → *X*′) ≤ *TE*(*Y* → *X*)

**Figure 9:**
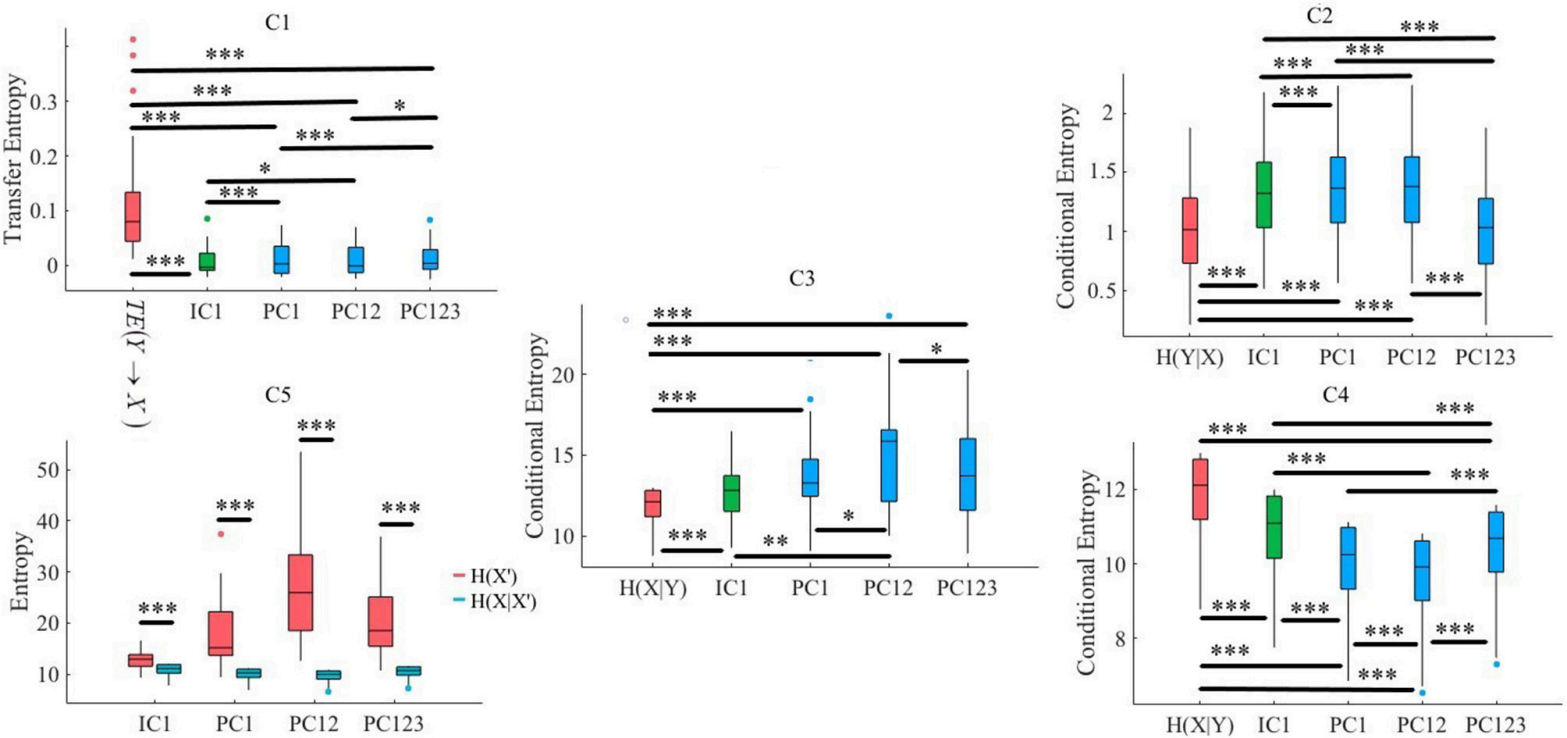
CTE. C1: Criterion 2.1: *TE*(*Y* → *X*′) ≤ *TE*(*Y* → *X*), C2: Criterion 2.2: *H*(*Y*|*X*) ≤ *H*(*Y*|*X*′), C3: Criterion 2.3: *H*(*X*|*Y*) ≤ *H*(*X*′|*Y*), C4: Criterion 2.4: *H*(*X*|*X*′) ≤ *H*(*X*|*Y*), C5: Criterion 2.5: *H*(*X*|*X*′) ≤ *H*(*X*′). In these subplots, IC1, PC1, PC12, and PC123 refer to the measured quantity by these components after their respective SBF attenuation algorithms. Asterisks mark the components with significant difference (^*^p < 0.05, ^**^p < 0.01, ^***^p < 0.001).

Kruskal-Wallis implied a significant effect of choice of components [*p* < 0.001, *H*_(4, 89)_ = 29.66, *r* = 0.57]. In addition, *post-hoc* comparison suggested that all components significantly reduced SBF effect in comparison with *TE*(*Y* → *X*) [PC1: *p* < 0.001, *W*_(34)_ = 3.72, *M* = 0.010, *SD* = 0.03, *r* = 0.62, PC12: *p* < 0.001, *W*_(34)_ = 3.72, *M* = 0.008, *SD* = 0.03, *r* = 0.62, PC123: *p* < 0.001, *W*_(34)_ = 3.72, *M* = 0.016, *SD* = 0.03, *r* = 0.62, IC1: < 0.001, *W*_(34)_ = 3.72, *M* = 0.017, *SD* = 0.03, *r* = 0.62]. On the other hand, whereas this comparison indicated non-significant difference between PC1 and PC12 [*p* = 0.95, *W*_(34)_ = 0.07, *r* = 0.01] as well as PC123 and IC1 [*p* = 0.65, *W*_(34)_ = 0.46, *r* = 0.08], we found significant differences in reduction of TE between PC1 and PC123 [*p* < 0.001, *W*_(34)_ = 3.42, *r* = 0.57], PC1 and IC1[*p* < 0.001, *W*_(34)_ = 3.68, *r* = 0.61], PC12 and PC123 [*p* < 0.05, *W*_(34)_ = 2.11, *r* = 0.35], as well as PC12 and IC1 [*p* < 0.03, *W*_(34)_ = 2.55, *r* = 0.43]. Figure 9, subplot C1, shows these results.

#### 3.3.2. Criterion 2.2: *H*(*Y*|*X*) ≤ *H*(*Y*|*X*′)

Kruskal-Wallis implied a significant effect of choice of components [*p* < 0.05, *H*_(4, 89)_ = 10.55, *r* = 0.34]. *Post-hoc* comparison implied a significant reduction of SBF-related information in frontal brain activity of participants by PC1 [*p* < 0.001, *W*_(34)_ = 3.72, *M*_*H*(*Y*|*X*)_ = 1.04, *SD*_*H*(*Y*|*X*)_ = 0.47, *M*_*PC*1_ = 1.31 *SD*_*PC*1_ = 0.47, *r* = 0.62], PC12 [*p* < 0.001, *W*_(34)_ = 3.72, *M*_*PC*12_ = 1.31, *SD*_*PC*12_ = 0.47, *r* = 0.69], and IC1 [*p* < 0.001, *W*_(34)_ = 3.72, *M*_*IC*1_ = 1.27, *SD*_*IC*1_ = 0.47, *r* = 0.69]. However, it indicated non-significant with respect to PC123 [*p* = 0.29, *W*_(34)_ = 1.07, *M*_*PC*123_ = 1.04, *SD*_*PC*123_ = 0.47, *r* = 0.18]. Moreover, this comparison indicated that adaptation of PC1 resulted in a significantly more reduced SBF-related information than PC123 [*p* < 0.001, *W*_(34)_ = 3.72, *r* = 0.69] as well as IC1 [*p* < 0.001, *W*_(34)_ = 3.72, *r* = 0.69]. Similarly, we found PC12 significantly more effective than PC123 [*p* < 0.001, *W*_(34)_ = 3.72, *r* = 0.69] and IC1 [*p* < 0.001, *W*_(34)_ = 3.72, *r* = 0.69]. Additionally, difference between IC1 and PC123 was significant [*p* < 0.001, *W*_(34)_ = 3.72, *r* = 0.69]. However, we found non-significant difference between PC1 and PC12 [*p* = 0.45, *W*_(34)_ = 0.76, *r* = 0.13]. Figure 9, subplot C2, shows these results.

#### 3.3.3. Criterion 2.3: *H*(*X*|*Y*) ≤ *H*(*X*′|*Y*)

We observed a significant effect of choice of components in resulting fNIRS data [*p* < 0.01, *H*_(4, 89)_ = 16.47, *r* = 0.51]. *Post-hoc* comparison suggested that all components were significantly effective in reducing the degree of correspondence between SBF and fNIRS time series of frontal brain activity [PC1: *p* < 0.001, *W*_(34)_ = 3.72, *M*_(*X*|*Y*)_ = 11.58, *SD*_(*X*|*Y*)_ = 1.53, *M*_*PC*1_ = 13.77, *SD*_*PC*1_ = 3.02, *r* = 0.69, PC12: *p* < 0.001, *W*_(34)_ = 3.72, *M*_*PC*12_ = 15.42, *SD*_*PC*12_ = 3.88, *r* = 0.69, PC123: *p* < 0.001, *W*_(34)_ = 3.72, *M*_*PC*123_ = 14.07, *SD*_*PC*123_ = 3.11, *r* = 0.69, IC1: *p* < 0.001, *W*_(34)_ = 3.72, *M*_*IC*1_ = 12.80, *SD*_*IC*1_ = 1.98, *r* = 0.69]. Additionally, this comparison implied that adaptation of PC12 yielded a significantly better performance than PC1 [*p* < 0.03, *W*_(34)_ = 2.20, *r* = 0.37], PC123 [*p* < 0.03, *W*_(34)_ = 2.46, *r* = 0.41], as well as IC1 [*p* < 0.01, *W*_(34)_ = 2.90, *r* = 0.48]. However, we found non-significant difference between PC1 and PC123 [*p* = 0.74, *W*_(34)_ = 0.33, *r* = 0.06], PC1 and IC1 [*p* = 0.20, *W*_(34)_ = 1.28, *r* = 0.21], as well as PC123 and IC1 [*p* = 0.17, *W*_(34)_ = 1.42, *r* = 0.24]. Figure 9, subplot C3, shows these results.

#### 3.3.4. Criterion 2.4: *H*(*X*|*X*′) ≤ *H*(*X*|*Y*)

Kruskal-Wallis indicated significant effect of choice of components [*p* < 0.001, *H*_(4, 89)_ = 23.52, *r* = 0.51]. *Post-hoc* comparison suggested that all components were significantly effective in retaining the correspondence between time series of frontal activity before and after application of SBF attenuation algorithms in comparison with SBF [PC1: *p* < 0.001, *W*_(34)_ = 3.72, *M*_*H*_(*X*|*Y*) = 11.58, *SD*_*H*_(*X*|*Y*) = 1.53, *M*_*PC*1_ = 9.72, *SD*_*PC*1_ = 1.56, *r* = 0.69, PC12: *p* < 0.001, *W*_(34)_ = 3.72, *M*_*PC*12_ = 9.40, *SD*_*PC*12_ = 1.55, *r* = 0.69, PC123: *p* < 0.001, *W*_(34)_ = 3.72, *M*_*PC*123_ = 10.17, *SD*_*PC*123_ = 1.55, *r* = 0.69, IC1: *p* < 0.001, *W*_(34)_ = 3.72, *M*_*IC*1_ = 10.57, *SD*_*IC*1_ = 1.55, *r* = 0.69]. Moreover, this comparison implied that PC12 resulted in significantly higher correspondence between time series of frontal brain activity before and after SBF attenuation than PC1 [*p* < 0.001, *W*_(34)_ = 3.72, *r* = 0.69], PC123 [*p* < 0.001, *W*_(34)_ = 4.24, *r* = 0.71], as well as IC1 [*p* < 0.001, *W*_(34)_ = 3.72, *r* = 0.69]. This was followed by significantly better performance by PC1 in contrast with PC123 [*p* < 0.001, *W*_(34)_ = 3.72, *r* = 0.69] and IC1 [*p* < 0.001, *W*_(34)_ = 3.72, *r* = 0.69]. Lastly, PC123 was significantly more effective than IC1 [*p* = *p* < 0.001, *W*_(34)_ = 3.72, *r* = 0.69]. Figure 9, subplot C4, shows these results.

#### 3.3.5. Criterion 2.5: *H*(*X*|*X*′) ≤ *H*(*X*′)

Pairwise Wilcoxon rank sum suggested that all components preserved information content of frontal activity [PC1: *p* < 0.001, *W*_(34)_ = 3.72, $M_{H\left(X|X^{\prime }\right)}$ = 9.72, ${SD}_{H\left(X|X^{\prime }\right)}$ = 1.56, $M_{H\left(X^{\prime }\right)}$ = 18.17, ${SD}_{H\left(X^{\prime }\right)}$ = 7.48, *r* = 0.69, PC12: *p* < 0.001, *W*_(34)_ = 3.72, $M_{H\left(X|X^{\prime }\right)}$ = 9.40, ${SD}_{H\left(X|X^{\prime }\right)}$ = 1.55, $M_{H\left(X^{\prime }\right)}$ = 27.66, ${SD}_{H\left(X^{\prime }\right)}$ = 11.71, *r* = 0.69 PC123: *p* < 0.001, *W*_(34)_ = -3.72, $M_{H\left(X|X^{\prime }\right)}$ = 10.17, ${SD}_{H\left(X|X^{\prime }\right)}$ = 1.55, $M_{H\left(X^{\prime }\right)}$ = 20.58, ${SD}_{H\left(X^{\prime }\right)}$ = 7.24, *r* = 0.88, IC1: *p* < 0.001, *W*_(34)_ = 3.72, $M_{H\left(X|X^{\prime }\right)}$ = 10.57, ${SD}_{H\left(X|X^{\prime }\right)}$ = 1.55 $M_{H\left(X^{\prime }\right)}$ = 12.84, ${SD}_{H\left(X^{\prime }\right)}$ = 2.00, *r* = 0.69]. Figure 9, subplot C5, depicts these results.

**Figure 10:**
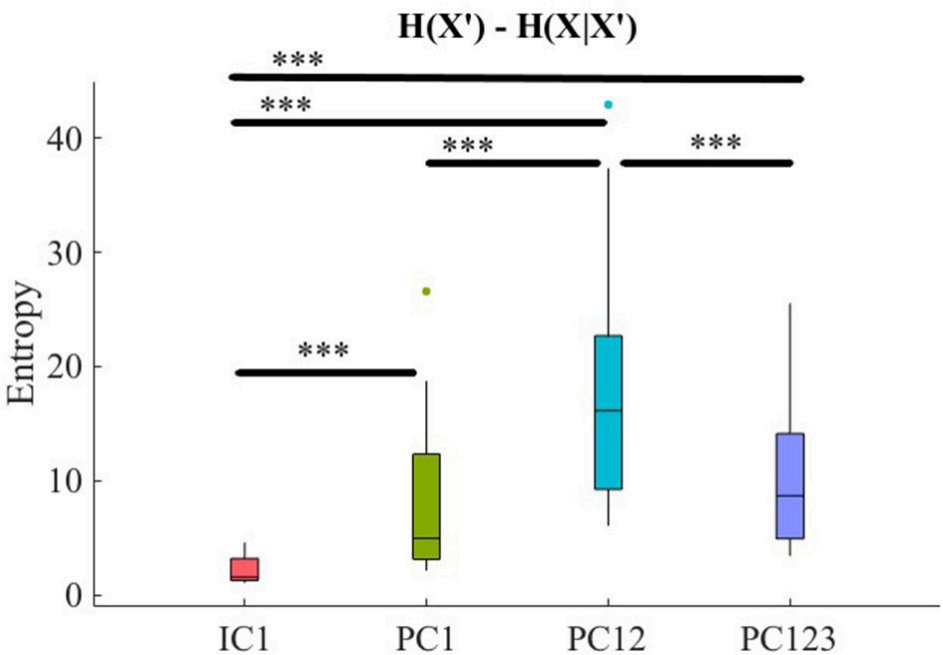
Comparison of maximal preservation of information content of frontal brain activity (i.e., *H*(*X*′) − *H*(*X*|*X*′) ≥ 0, Criterion 2.5) after application of PCA- and ICA-based SBF attenuation algorithms on CTE. Asterisks mark the components with significant difference (^*^p < 0.05, ^**^p < 0.01, ^***^p < 0.001).

Kruskal-Wallis test indicated a significant effect of choice of component on preservation of information content of fNIRS time series [i.e., *H*(*X*′) − *H*(*X*|*X*′) ≥ 0] [*p* < 0.01, *H*_(3, 71)_ = 11.15, *r* = 0.39]. *Post-hoc* comparison implied significantly higher preservation of information content of frontal brain activity with respect to PC12 in comparison with PC1 [*p* < 0.001, *W*_(34)_ = 3.51, *M*_*PC*12_ = 18.27, *SD*_*PC*12_ = 10.81, *M*_*PC*1_ = 8.45, *SD*_*PC*1_ = 7.04], *r* = 0.59, PC123 [*p* < 0.001, *W*_(34)_ = 3.72, *M*_*PC*123_ = 10.41, *SD*_*PC*123_ = 6.45, *r* = 0.62], as well as IC1 [*p* < 0.001, *W*_(34)_ = 3.72, *M*_*IC*1_ = 2.27, *SD*_*IC*1_ = 1.20, *r* = 0.62]. Furthermore, PC1 was significantly more effective than IC1 [*p* < 0.001, *W*_(34)_ = 3.33, *r* = 0.56]. Lastly, we found PC123 significantly better than IC1 [< 0.001, *W*_(34)_ = 3.68, *r* = 0.61]. However, this test implied that difference between PC1 and PC123 was non-significant [*p* = 0.29, *W*_(34)_ = 1.07, *r* = 0.18]. Figure 10 shows these results.

### 3.4. LMT

**Figure 11:**
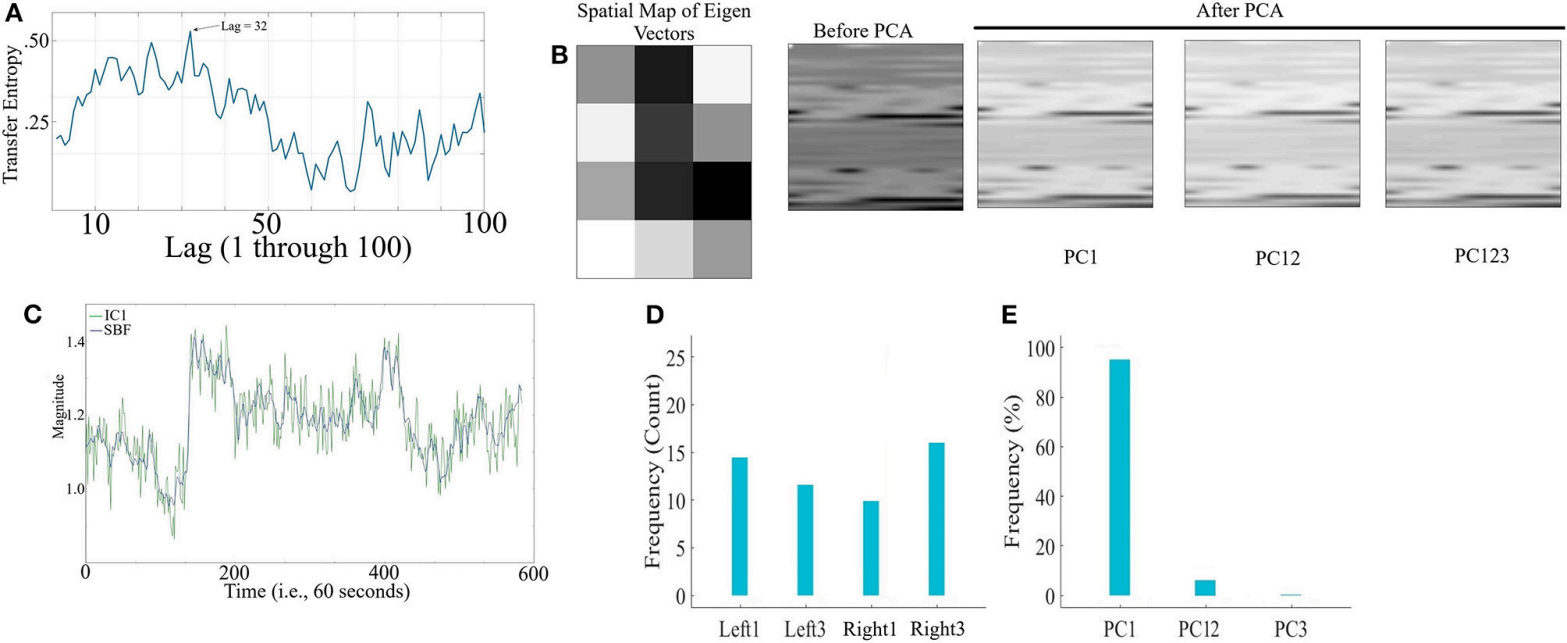
LMT - **(A)** Grand-average of TE values for Lag = 1, …, 100 (i.e., up to 10 s of lag, considering the sampling rate of 10.0 Hz in our device). Maximum TE was at Lag = 32 (equivalent to time = 3.2 s) **(B)** Sample spatial map of eigenvectors by PCA-based SBF attenuation algorithm along with a sample participant's PFC time series (in matrix forms for better visualization purpose) before and after application of PC1, PC12, and PC123 components. Distinctive effect of these components on participant's PFC time series is evident in this subplot. All matrices are scaled within [0, 1] interval for ease of comparison. **(C)** Sample SBF (blue) and the corresponding IC1 component computed by ICA-based SBF attenuation algorithm (green). The depicted IC1 component pertains to the case in which *Right*_1_ was selected as component with highest coefficient of spatial uniformity. **(D)** Frequency of selected channels as a component with highest coefficient of spatial uniformity by ICA-based SBF attenuation algorithm for LMT dataset. **(E)** Percentages of the variance-explained by each of the principal components (i.e., PC1, PC12, PC123) of PCA-based SBF attenuation algorithm for LMT dataset.

Figure 11A corresponds to the grand-average of the computed TE values for lags 1 through 100 (i.e., up to 10 s of lag, considering the sampling rate of 10.0 Hz of our device) in LMT. This subplot indicates that maximum SBF transferred information was, on average, at lag = 32 (i.e., 3.2 s). This was the lag we used during our analyses. Wilcoxon rank sum test implied significant effect of SBF on fNIRS time series of frontal brain activity of participants while performing VFT working memory task [*p* < 0.001, *W*_(25)_ = 4.46, *M* = 0.58, *SD* = 0.19, *r* = 0.70].

Figure 11B visualizes a sample spatial map of eigenvectors by PCA-based SBF attenuation algorithm along with a sample participant's PFC time series (in matrix forms for better visualization purpose) before and after application of PC1, PC12, and PC123 components. Distinctive effect of these components on participant's PFC time series is evident in this subplot. All matrices are scaled within [0, 1] interval for ease of comparison. We observed significant differences in PFC time series between before and after application of PC1 [*p* < 0.001, *W*_(3, 358)_ = 7.70, *r* = 0.13, *M*_*Before*_ = 216.53, *SD*_*Before*_ = 32.81, *M*_*PC*1_ = 211.56, *SD*_*PC*1_ = 19.21], PC12 [*p* < 0.001, *W*_(3, 358)_ = 9.03, *r* = 0.16, *M*_*PC*12_ = 210.53, *SD*_*PC*12_ = 13.97], and PC123 [*p* < 0.001, *W*_(3, 358)_ = 6.34, *r* = 0.11, *M*_*PC*123_ = 212.60, *SD*_*PC*123_ = 12.86].

Figure 11C shows a sample SBF (blue) along with the computed IC1 by ICA-based SBF attenuation algorithm (green) (pertinent to the case in which *Right*_1_ was selected as the component with highest coefficient of spatial uniformity). Although this algorithm resulted in selection of different channels as the component with highest coefficient of spatial uniformity (Figure 11D, *Left*_1_ = 26.92%, *Left*_3_ = 23.08%, *Right*_1_ = 30.77%, *Right*_3_ = 19.23%), these components were significantly correlated with simulated noise (*Left*_1_: 0.77, *p* < 0.001, *Left*_3_: *r* = 0.73, *p* < 0.001, *Right*_1_: *r* = 0.70, *p* < 0.001, and *Right*_3_: *r* = 0.76, *p* < 0.001). On the other hand, PCA-based algorithm (Figure 11E) exhibited a higher specificity in selecting the principal components (PC1 = 94.53%, PC12 = 5.11%, PC123 = 0.36%).

In what follows, we examine the effectiveness of PCA- and ICA-based SBF attenuation algorithms on reduction of observed impact of SBF on fNIRS frontal brain activity as well as their utility in preservation of information content of this activity in resulting fNIRS time series through investigation of Criterion 2.1 through Criterion 2.5.

#### 3.4.1. Criterion 2.1: *TE*(*Y* → *X*′) ≤ *TE*(*Y* → *X*)

**Figure 12:**
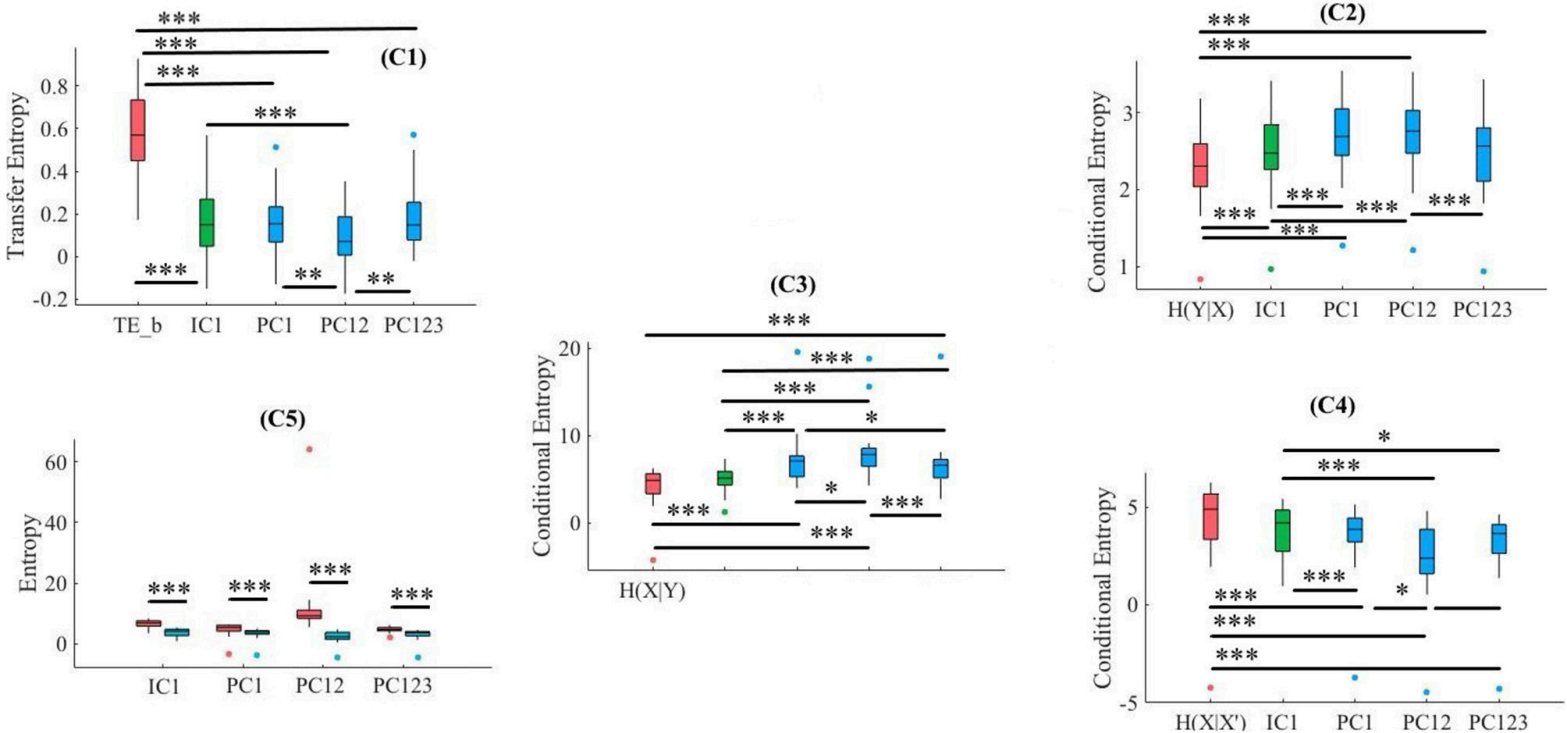
LMT. C1: Criterion 2.1: *TE*(*Y* → *X*′) ≤ *TE*(*Y* → *X*), C2: Criterion 2.2: *H*(*Y*|*X*) ≤ *H*(*Y*|*X*′), C3: Criterion 2.3: *H*(*X*|*Y*) ≤ *H*(*X*′|*Y*), C4: Criterion 2.4: *H*(*X*|*X*′) ≤ *H*(*X*|*Y*), C5: Criterion 2.5: *H*(*X*|*X*′) ≤ *H*(*X*′). In these subplots, IC1, PC1, PC12, and PC123 refer to the measured quantity by these components after their respective SBF attenuation algorithms. Asterisks mark the components with significant difference (^*^p < 0.05, ^**^p < 0.01, ^***^p < 0.001).

Kruskal-Wallis test implied significant effect of choice of components [*p* < 0.001, *H*_(4, 129)_ = 56.39, *r* = 0.66]. Moreover, *post-hoc* comparison implied that all components significantly reduced SBF effect in comparison with *TE*(*Y* → *X*) [PC1: *p* < 0.001, *W*_(50)_ = 4.46, *M* = 0.16, *SD* = 0.15, *r* = 0.62, PC12: *p* < 0.001, *W*_(50)_ = 4.43, *M* = 0.08, *SD* = 0.13, *r* = 0.61, PC123:*p* < 0.001, *W*_(50)_ = 4.46, *M* = 0.18, *SD* = 0.16, *r* = 0.62, IC1: *p* < 0.001, *W*_(50)_ = 4.43, *M* = 0.18, *SD* = 0.17, *r* = 0.61]. *Post-hoc* comparison indicated that PC12 significantly performed better in attenuation of the effect of SBF than PC1 [*p* < 0.01, *W*_(50)_ = 2.73, *r* = 0.38], PC123 [*p* < 0.01, *W*_(50)_ = 3.04, *r* = 0.42], and IC1 [*p* < 0.001, *W*_(50)_ = 3.34, *r* = 0.46]. On the other hand, we observed non-significant differences between PC1 and PC123 [*p* = 0.36, *W*_(50)_ = 0.93, *r* = 0.13], PC1 and IC1 [*p* = 0.47, *W*_(50)_ = 0.72, *r* = 0.10], as well as PC123 and IC1 [*p* = 0.75, *W*_(50)_ = 0.32, *r* = 0.04]. Figure 12, subplot C1, illustrates these results.

#### 3.4.2. Criterion 2.2: *H*(*Y*|*X*) ≤ *H*(*Y*|*X*′)

Kruskal-Wallis implied a significant effect of choice of components [*p* < 0.01, *H*_(4, 129)_ = 13.32, *r* = 0.32]. *Post-hoc* comparison indicated a significant reduction of SBF-related information in frontal brain activity of participants by PC1 [*p* < 0.001, *W*_(50)_ = 4.46, *M*_*H*(*Y*|*X*)_ = 2.31, *SD*_*H*(*Y*|*X*)_ = 0.50, *M*_*PC*1_ = 2.69, *SD*_*PC*1_= 0.48, *r* = 0.62], PC12 [*p* < 0.001, *W*_(50)_ = 4.46, *M*_*PC*12_ = 2.71, *SD*_*PC*12_ = 0.50, *r* = 0.62], PC123 [*p* < 0.001, *W*_(50)_ = 3.95, *M*_*PC*123_ = 2.49, *SD*_*PC*123_ = 0.53, *r* = 0.55], and IC1 [*p* < 0.001, *W*_(50)_ = 4.46, *M*_*IC*1_ = 2.47, *SD*_*IC*1_ = 0.53, *r* = 0.62]. Additionally, this comparison indicated that adaptation of PC1 resulted in a significantly more reduced SBF-related information than PC123 [*p* < 0.001, *W*_(50)_ = 4.23, *r* = 0.49] as well as IC1 [*p* < 0.001, *W*_(50)_ = 4.33, *r* = 0.49]. Similarly, we found PC12 significantly more effective than PC123 [*p* < 0.001, *W*_(50)_ = 4.10, *r* = 0.49] and IC1 [*p* < 0.001, *W*_(50)_ = 4.36, *r* = 0.49]. However, we found non-significant differences between PC1 and PC12 [*p* = 0.49, *W*_(50)_ = 0.70, *r* = 0.10] as well as IC1 and PC123 was significant [*p* = 0.14, *W*_(50)_ = 1.49, *r* = 0.21]. Figure 12, subplot C2, shows these results.

#### 3.4.3. Criterion 2.3: *H*(*X*|*Y*) ≤ *H*(*X*′|*Y*)

Kruskal-Wallis indicated a significant effect of choice of components in resulting fNIRS data [*p* < 0.001, *H*_(4, 129)_ = 47.41, *r* = 0.61]. *Post-hoc* comparison suggested that all components were significantly effective in reducing the degree of correspondence between SBF and fNIRS time series of frontal brain activity [PC1: *p* < 0.001, *W*_(50)_ = 4.46, *M*_*H*(*X*|*Y*)_ = 4.22, *SD*_*H*(*X*|*Y*)_ = 2.21, *M*_*PC*1_ = 7.11, *SD*_*PC*1_ = 3.03, *r* = 0.62, PC12: *p* < 0.001, *W*_(50)_ = 4.46, *M*_*PC*12_ = 8.17, *SD*_*PC*12_ = 3.01, *r* = 0.62, PC123: *p* < 0.001, *W*_(50)_ = 4.46, *M*_*PC*123_ = 6.57, *SD*_*PC*123_ = 2.96, *r* = 0.62]. However, we observed that the effect of IC1 was non-significant [IC1: *p* = 0.10, *W*_(50)_ = 1.66, *M*_*IC*1_ = 5.09, *SD*_*IC*1_ = 1.36, *r* = 0.23]. In addition, this comparison indicated that adaptation of PC12 yielded a significantly better performance than PC1 [*p* < 0.03, *W*_(50)_ = 2.30, *r* = 0.32], PC123 [*p* < 0.001, *W*_(50)_ = 3.42, *r* = 0.47], as well as IC1 [*p* < 0.001, *W*_(50)_ = 4.46, *r* = 0.62]. Moreover, PC1 was significantly more effective than PC123 [*p* < 0.05, *W*_(50)_ = 1.97, *r* = 0.27] and IC1 [*p* < 0.001, *W*_(50)_ = 3.95, *r* = 0.55]. Additionally, PC123 was significantly different from IC1 [*p* < 0.001, *W*_(50)_ = 2.58, *r* = 0.36]. Figure 12, subplot C3, plots these results.

#### 3.4.4. Criterion 2.4: *H*(*X*|*X*′) ≤ *H*(*X*|*Y*)

Kruskal-Wallis indicated a significant effect of choice of components [*p* < 0.001, *H*_(4, 129)_ = 30.93, *r* = 0.49]. *Post-hoc* comparison suggested that all components were significantly effective in retaining the correspondence between time series of frontal activity before and after application of SBF attenuation [PC1: *p* < 0.001, *W*_(50)_ = 3.24, *M*_*H*(*X*|*Y*)_ = 4.22, *SD*_*H*(*X*|*Y*)_ = 2.22, *M*_*PC*1_ = 2.71, *SD*_*PC*1_ = 1.72, *r* = 0.45, PC12:*p* < 0.001, *W*_(50)_ = 4.46, *M*_*PC*12_ = 1.97, *SD*_*PC*12_ = 1.88, *r* = 0.62, PC123: *p* < 0.001, *W*_(50)_ = 3.39, *M*_*PC*123_ = 2.71, *SD*_*PC*123_ = 1.74, *r* = 0.47] However, we observed that the effect of IC1 was non-significant [*p* = 0.13, *W*_(50)_ = 1.51, *M*_*IC*1_ = 3.67, *SD*_*IC*1_ = 1.47, *r* = 0.21]. Additionally, this comparison implied that PC12 resulted in significantly higher correspondence between time series of frontal brain activity before and after SBF attenuation than PC1 [*p* < 0.03, *W*_(50)_ = 2.07, *r* = 0.29], PC123 [*p* < 0.001, *W*_(50)_ = 2.20, *r* = 0.31], as well as IC1 [*p* < 0.001, *W*_(50)_ = 3.85, *r* = 0.53]. Although PC1 performed significantly better than IC1 [*p* < 0.001, *W*_(50)_ = 2.22, *r* = 0.31], its difference with PC123 was non-significant [*p* = 0.27, *W*_(50)_ = 1.10, *r* = 0.15]. Lastly, PC123 was significantly more effective than IC1 [*p* = *p* < 0.03, *W*_(50)_ = 2.30, *r* = 0.32]. Figure 12, subplot C4, shows these results.

#### 3.4.5. Criterion 2.5: *H*(*X*|*X*′) ≤ *H*(*X*′)

Pairwise Wilcoxon rank sum suggested that all components preserved information content of frontal activity [PC1: *p* < 0.001 *W*_(50)_ = 3.67, $M_{H\left(X|X^{\prime }\right)}$ = 2.71, ${SD}_{H\left(X|X^{\prime }\right)}$ = 1.72, $M_{H\left(X^{\prime }\right)}$ = 4.80, ${SD}_{H\left(X^{\prime }\right)}$ = 2.11, *r* = 0.51, PC12:*p* < 0.001, *W*_(50)_ = 4.46, $M_{H\left(X|X^{\prime }\right)}$ = 1.97, ${SD}_{H\left(X|X^{\prime }\right)}$ = 1.88, $M_{H\left(X^{\prime }\right)}$ = 11.55, ${SD}_{H\left(X^{\prime }\right)}$ = 10.94, *r* = 0.62, PC123: *p* < 0.001, *W*_(50)_ = 4.38, $M_{H\left(X|X^{\prime }\right)}$ = 2.713, ${SD}_{H\left(X|X^{\prime }\right)}$ = 1.74, $M_{H\left(X^{\prime }\right)}$ = 4.83, ${SD}_{H\left(X^{\prime }\right)}$ = 0.91, *r* = 0.61, IC1: *p* < 0.001, *W*_(50)_ = 4.46, $M_{H\left(X|X^{\prime }\right)}$ = 3.67, ${SD}_{H\left(X|X^{\prime }\right)}$ = 1.47, $M_{H\left(X^{\prime }\right)}$ = 6.67, ${SD}_{H\left(X^{\prime }\right)}$ = 1.40, *r* = 0.62]. Figure 12, subplot C5, depicts these results.

**Figure 13:**
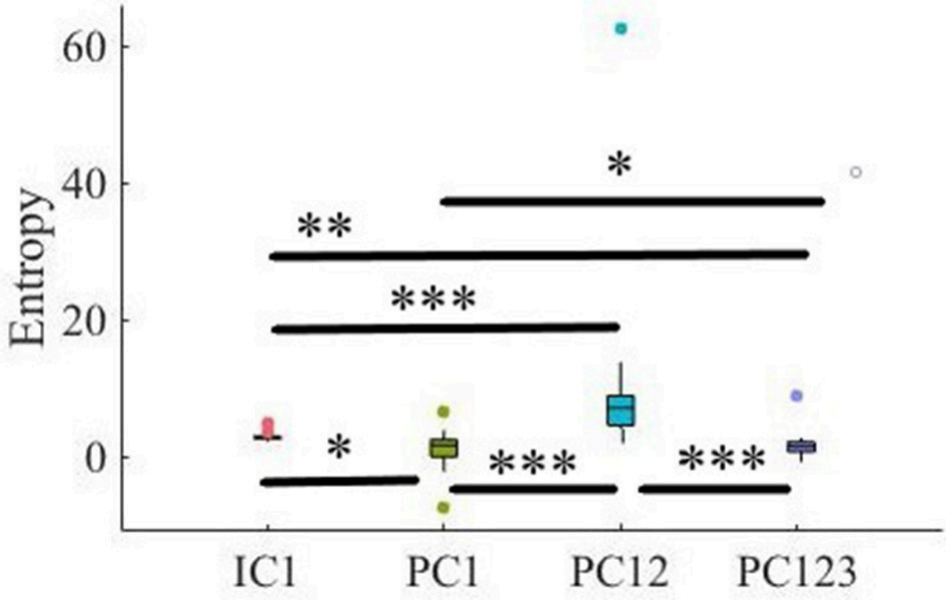
Comparison of maximal preservation of information content of frontal brain activity (i.e., *H*(*X*′) − *H*(*X*|*X*′) ≥ 0, Criterion 2.5) after application of PCA- and ICA-based SBF attenuation algorithms on LMT. Asterisks mark the components with significant difference (^*^p < 0.05, ^**^p < 0.01, ^***^p < 0.001).

Kruskal-Wallis test indicated a significant effect of choice of component on preservation of information content of fNIRS time series [i.e., *H*(*X*′) − *H*(*X*|*X*′) ≥ 0] [*p* < 0.01, *H*_(3, 103)_ = 16.01, *r* = 0.16]. *Post-hoc* paired comparison implied a significantly higher preservation of information content of frontal brain activity with respect to PC12 in comparison with PC1 [*p* < 0.001, *W*_(50)_ = 4.30, *M*_*PC*1_ = 2.10, *SD*_*PC*1_ = 2.46, *M*_*PC*12_ = 9.58, *SD*_*PC*12_ = 11.29, *r* = 0.60], PC123 [*p* < 0.001, *W*_(50)_ = 4.43, *M*_*PC*123_ = 2.12, *SD*_*PC*123_ = 1.77, *r* = 0.61], as well as IC1 [*p* < 0.001, *W*_(50)_ = 4.43, *M*_*IC*1_ = 2.10, *SD*_*IC*1_ = 0.51, *r* = 0.61]. IC1 was significantly more effective than and PC1 [*p* < 0.03, *W*_(50)_ = 2.25, *r* = 0.31] and PC123 [*p* < 0.01, *W*_(50)_ = 3.19, *r* = 0.44]. Last, we found that PC1 was significantly more effective than PC123 [*p* < 0.03, *W*_(50)_ = 2.41, *r* = 0.33]. Figure 13 shows these results.

## 4. Discussion

In this article, we pinpointed the lack of perspective in previous studies on frontal activity's information preservation while attenuating the effect of SBF on fNIRS time series. Subsequently, we proposed five information-theoretic criteria for quantification of the frontal activity's information preservation during SBF attenuation process. We utilized the concept of TE (Schreiber, 2000) to quantify the effect of SBF on fNIRS time series of frontal brain activity via transferring undesired information onto fNIRS measurement. Advantages of TE for this purpose are twofold: First, TE, similar to MI and unlike other correlation measures, is always ≥0 that makes it a good quantitative metric for amount of information transferred/shared. Second, TE, unlike MI and other correlation measures, has a direction [i.e., TE(X ⇒ Y) ≠ TE(Y ⇒ X)] which allows for causal reasoning about which process induces the observed effect on transferred/shared information. Additionally, we exploited the concept of MI and its correspondence with conditional entropy between interacting continuous random variables (Cover and Thomas, 2006; Stone, 2015) to formalize criteria for frontal brain activity's information preservation in resulting fNIRS time series once the process of SBF attenuation is complete.

We verified the validity our criteria on PCA- (Zhang et al., 2005) and ICA-based (Kohno et al., 2007) SBF attenuation algorithms using simulated time series with additive Gaussian noise. We chose these SBF attenuation algorithms due to their widespread adaptation in recent literature (Katura et al., 2008; Kiguchi and Funane, 2014; Tak and Ye, 2014; Naseer and Hong, 2015; Sato et al., 2016; Zhang et al., 2016). Although these algorithms make assumption on orthogonality and statistical independence of their components, their well-defined mathematical formulation help eliminate further empirical assumptions on causal and/or explanatory effects (e.g., channels with short source-detector distances as representatives of SBF, etc.). Subsequently, we examined our criteria on two different Working Memory (WM) tasks and a naturalistic conversational settings. Our results implied a significant effect of SBF on fNIRS time series of frontal brain activity through transfer of undesired information. This finding that was founded on analysis of the information flow from SBF onto fNIRS time series of frontal brain activity presented a systematic approach to quantification of the SBF as an interfering process during fNIRS measurement, thereby drawing an informed conclusion on this issue (Takahashi et al., 2011; Sato et al., 2013).

In addition, our results implied that mere reduction of SBF influence on fNIRS time series of frontal activity was insufficient to warrant frontal activity's information preservation. More importantly, we found this observation to hold true, irrespective of the nature of the adapted task or age of the participants. This, in turn, provided further support for inefficiency of such measures as correlation coefficient, signal-to-noise ratio, or Pearson *R*^2^ (Zhang et al., 2005; Kohno et al., 2007; Gagnon et al., 2011) in determination of the significance of the reduced effect of SBF due to their inability in detecting the direction of the information flow (i.e., causality) between interacting processes (Kinney and Atwal, 2014).

Moreover, our results implied a higher fidelity of PCA-based algorithm in preservation of information content of frontal brain activity in comparison with ICA-based approach. This observation was in accord with the findings by Sato et al. (2016). Furthermore, our results revealed a substantial effect of selected number of components on performance of PCA-based algorithm. Concretely, they indicated that combination of first two principal components of PCA-based algorithm resulted in most efficient SBF attenuation while ensuring a significantly higher (i.e., in comparison with other adapted components) frontal activity's information preservation. This provided an evidence for a reliable choice among existing SBF attenuation algorithms and their inconclusive number of components (Zhang et al., 2005; Sato et al., 2016) to ensure minimum loss of frontal activity's information content during SBF attenuation process.

Further evidence on substantial difference in performance of these algorithms was due to the high variability of ICA-based SBF attenuation in determination of the component with highest coefficient of spatial uniformity. This variability that was originally reported by Kohno et al. (2007) reduces the reliability of this algorithm, given its contingency in selecting the channel of interest (e.g., long source-detector distance channels). On the other hand, PCA-based SBF attenuation algorithm exhibited a stable distribution of the percentages of the variance-explained among its selected components. It is worth noting that observed distribution of these components in our results is in close correspondence with Sato et al. (2016), although their subtle differences is appreciated in light of comparably larger number of channels in their study. Considering these observations, it is apparent that such differences in stability of these algorithms in determination of their respective SBF-related component(s) impose substantial variation in analyses results. Given the observed variability in choice of components, as indicated by our results on simulated as well as real time data and irrespective of the age of participants, it is apparent that an SBF algorithm with the stable choice of component(s) to reduce the effect of SBF to retain the information content pertinent to brain activity of human subjects is highly desirable. Our results suggested that ICA algorithm falls short in achieving this objective.

Taken together, we provided evidence that lack of perspective on preservation of information content of frontal brain activity during SBF attenuation underlies the contrasting findings with inconclusive results in previous studies. We showed that a mere reduction of SBF influence on fNIRS time series of frontal activity is insufficient in warranting frontal activity's information preservation in resulting fNIRS time series data. Subsequently, we showed a higher fidelity of PCA-based algorithm in achieving this information preservation in comparison with ICA-based approach. Lastly, we showed that combination of first two components of PCA-based algorithm resulted in most information preservation while ensuring significant attenuation of SBF effect. Our findings contribute to the field by presenting a systematic approach to quantification of the SBF as an interfering process during fNIRS measurement, thereby drawing an informed conclusion on this debate (Takahashi et al., 2011; Sato et al., 2013). Furthermore, they provide evidence for a reliable choice among existing SBF attenuation algorithms and their inconclusive number of components (Zhang et al., 2005; Sato et al., 2016), thereby ensuring minimum loss of information content of fNIRS cortical activity through SBF attenuation process.

## Ethics Statement

This study was carried out in accordance with the recommendations of the ethical committee of Advanced Telecommunications Research Institute International (ATR) with written informed consent from all subjects. All subjects gave written informed consent in accordance with the Declaration of Helsinki. The protocol was approved by the ATR ethical committee (approval code:16-601-1).

## Author Contributions

SK proposed the information-theoretic criteria and carried out the analyses. HS acted as research lead, designing the experiments, supervising their progress, and taking part in experimental setups during data collections. MO conducted the experiments and performed data collection. As the head of Hiroshi Ishiguro Laboratories (HIL), HI oversaw the entire activity of all research teams and themes, ensuring the soundness of all proposals, quality of results, and their validity. SK and HS contributed equally in preparation of the manuscript.

### Conflict of Interest Statement

The authors declare that the research was conducted in the absence of any commercial or financial relationships that could be construed as a potential conflict of interest.

## Footnotes

^1^X → Y → Z if and only if X and Z are conditionally independent, given Y i.e., they form a Markov chain Cover and Thomas (2006, p. 34).

^2^Continuous random variables can have negative entropy. For instance, *H*(*X*) < 0 if $\sigma_{X}^{2}<\frac{1}{2\pi e}$ or $\mu_{X}<\frac{1}{e}$ in case of normal and Poisson distributions.

^3^If *H*(*X*|*Y*) ≤ 0 in Equation (18), indicating the possibility for *H*(*X*|*Y*) = 0, then *H*(*X*|*Y*) = 0 ⇒ *H*(*X*|*Y*) = *H*(*X*|*C*) ⇒ *Y* = *C* which contradicts *X* = *C* + *Y*.
