# Supplementary material for: An Information-Theoretic Approach to Quantitative Analysis of the Correspondence Between Skin Blood Flow and Functional Near-Infrared Spectroscopy Measurement in Prefrontal Cortex Activity
Source: Front Neurosci. 2019 Feb 15;13:79. doi: 10.3389/fnins.2019.00079 (PMC6384277; doi:10.3389/fnins.2019.00079)
Supplement: Supplementary file 1 [file Data_Sheet_1.pdf]

# Supplementary Materials:
# An Information-Theoretic Approach to Quantitative Analysis of Correspondence between Skin Blood Flow and Functional Near-Infrared Spectroscopy Measurement in Prefrontal Cortex Activity

**Soheil Keshmiri [1,*], Hidenubo Sumioka [1], Masataka Okubo [1] Hiroshi Ishiguro [1,2]**

[1] *Hiroshi Ishiguro Laboratories (HIL), Advanced Telecommunications Research Institute International (ATR), Kyoto, Japan*
[2] *Graduate School of Engineering Science, Osaka University, Japan*

Correspondence*:
Corresponding Author
soheil@atr.jp

## 1 ANALYSIS RESULTS OF THE RIGHT HEMISPHERIC FRONTAL CORTEX

### 1.1 VFT

Wilcoxon rank sum test implied significant effect of SBF on NIRS time series of frontal brain activity of participants while performing VFT working memory task (p $<$ .001, W(125) = 7.82, M = .44, SD = .15, r = .70). In what follows, we examine the effectiveness of PCA- and ICA-based SBF attenuation algorithms on reduction of observed impact of SBF on NIRS frontal brain activity as well as their utility in preservation of information content of this activity in resulting NIRS time series through investigation of Criterion 2.1 through Criterion 2.5.

#### 1.1.1 Criterion 2.1: $\boldsymbol{TE(Y \rightarrow X') \leq TE(Y \rightarrow X)}$

Kruskal-Wallis test implied significant effect of choice of components (p $<$ .001, H(4, 629) = 237.34, r = .61). Moreover, post hoc comparison implied that all components significantly reduced SBF effect in comparison with $TE(Y \rightarrow X)$ (PC1: p $<$ .001, W(250) = 7.81, M = .01, SD= .03, r = .49, PC12: p $<$ .001, W(250) = 7.82, M = .009, SD = .03, r = .49, PC123: p $<$ .001, W(250) = 7.82, M = .05, SD = .03, r = .49, IC1: p $<$ .001, W(250) = 7.82, M = .04, SD = .03, r = .49). On the other hand, whereas this comparison indicated non-significant difference between PC1 and PC12 (p = 1.00, W(250) = .002, r = 0.0) as well as PC123 and IC1 (p = .43, W(250) = .84, r = .05), we found significant differences in reduction of TE between PC1 and PC123 (p $<$ .001, W(250) = 6.38, r = .40), PC1 and IC1(p $<$ .001, W(250) = 6.90, r = .43), PC12 and PC123 (p = $<$ .001, W(250) = 6.64, r = .42), as well as PC12 and IC1 (p $<$ .001, W(250) = 6.54, r = .41). Figure 1, subplot C1, illustrates these results.

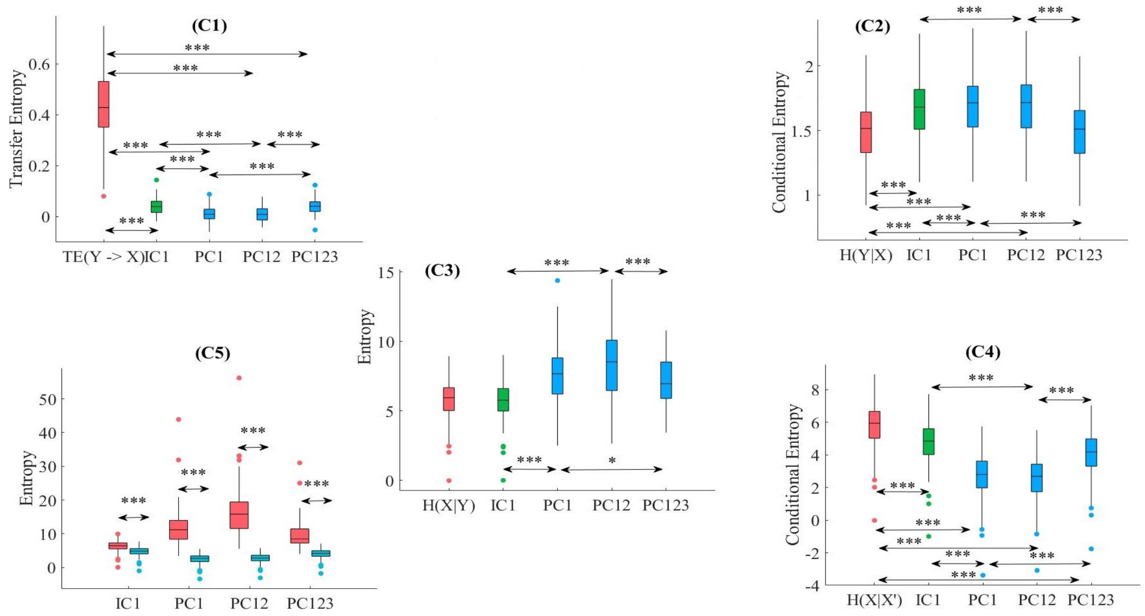


**Figure 1.** VFT - C1: Criterion 2.1: $TE(Y \rightarrow X') \leq TE(Y \rightarrow X)$, C2: Criterion 2.2: $H(Y|X) \leq H(Y|X')$, C3: Criterion 2.3: $H(X|Y) \leq H(X'|Y)$, C4: Criterion 2.4: $H(X|X') \leq H(X|Y)$, C5: Criterion 2.5: $H(X|X') \leq H(X')$. In these subplots, IC1, PC1, PC12, and PC123 refer to the measured quantity by these components after their respective SBF attenuation algorithms. Asterisks mark the components with significant difference (*: p < .05, **: p < .01, ***: p < .001).

### 1.1.2 Criterion 2.2: $H(Y|X) \leq H(Y|X')$

Kruskal-Wallis implied a significant effect of choice of components (p < .001, H(4, 629) = 47.36, r = .27). Post hoc comparison indicated a significant reduction of SBF-related information in frontal brain activity of participants by PC1 (p < .001, W(250) = 7.82, $M_{H(Y|X)}$ = 1.53, $SD_{H(Y|X)}$ = .45, $M_{PC1}$ = 1.62, $SD_{PC1}$ = .51, r = .49), PC12 (p < .001, W(250) = 7.81, $M_{PC12}$ = 1.62, $SD_{PC12}$ = .50, r = .49), and IC1 (p < .001, W(250) = 7.81, $M_{IC1}$ = 1.56 $SD_{IC1}$ = .53, r = .49). However, it was non-significant with respect to PC123 (p = .20, W(250) = 1.28, $M_{PC123}$ = 1.53, $SD_{PC1}$ = .50, r = .08). Additionally, this comparison indicated that adaptation of PC1 resulted in a significantly more reduced SBF-related information than PC123 (p = .19, W(250) = 1.28, $M_{PC1}$ = -1.52, $SD_{PC1}$ = .49, r = .08) as well as IC1 (p < .001, W(250) = 7.79, r = .49). Similarly, we found PC12 significantly more effective than PC123 (p < .001, W(250) = 7.82, r = .49) and IC1 (p < .001, W(250) = 7.54, r = .49). Additionally, difference between IC1 and PC123 was significant (p < .001, W(250) = 7.81, r = .49). However, we found non-significant difference between PC1 and PC12 (p = .15, W(250) = 1.46, r = .09). Figure 1, subplot C2, shows these results.

### 1.1.3 Criterion 2.3: $H(X|Y) \leq H(X'|Y)$

Kruskal-Wallis indicated a significant effect of choice of components in resulting NIRS data (p < .001, H(4, 629) = 83.87, r = .36). Post hoc comparison suggested that all components were significantly effective in reducing the degree of correspondence between SBF and NIRS time series of frontal brain activity (PC1: p < .001, W(250) = 7.62, $M_{H(X|Y)}$ = 5.94, $SD_{H(X|Y)}$ = 1.67, $M_{PC1}$= 7.65, $SD_{PC1}$ = 2.02, r = .48, PC12: p < .001, W(250) = 7.82, $M_{PC12}$ = 8.37, $SD_{PC12}$ = 2.28, r = .49, PC123: p < .001, W(250) = 7.82, $M_{PC123}$ = 7.14, $SD_{PC123}$ = 1.75, r = .49, IC1: p < .001, W(250) = 3.90, $M_{IC1}$ = 5.81, $SD_{IC1}$ = 1.63, r = .25). In addition, this comparison indicated that adaptation of PC12 yielded a significantly better performance than PC1 (p < .001, W(250) = 5.12, r = .32), PC123 (p < .001, W(250) = 5.21, r = .33),

as well as IC1 ($p < .001$, W(250) = 7.81, r = .49). Moreover, PC1 was significantly more effective than PC123 ($p < .01$, W(250) = 2.92, r = .18) and IC1 ($p < .001$, W(250) = 7.53, r = .47). Additionally, PC123 was significantly different from IC1 ($p < .001$, W(250) = 7.65, r = .48). Figure 1, subplot C3, plots these results.

### 1.1.4 Criterion 2.4: $H(X|X') \leq H(X|Y)$

Kruskal-Wallis indicated a significant effect of choice of components ($p < .001$, H(4, 629) = 136.57, r = .47). Post hoc comparison suggested that all components were significantly effective in retaining the correspondence between time series of frontal activity before and after application of SBF attenuation (PC1: $p < .001$, W(250) = 6.88, $M_{PC1}$ = 3.71, $SD_{PC1}$ = 1.63, r = .48, PC12: $p < .001$, W(250) = 7.63, $M_{H(X|Y)}$ = 5.94, $SD_{H(X|Y)}$ = 1.67, $M_{PC12}$ = 2.63, $SD_{PC12}$ = 1.64, r = .48, PC123: $p < .001$, W(250) = 6.63, $M_{PC123}$ = 3.81, $SD_{PC123}$ = 1.65, r = .35, IC1: $p < .001$, W(250) = 4.40, $M_{IC1}$ = 4.86, $SD_{IC1}$ = 1.61, r = .28). Additionally, this comparison implied that PC12 resulted in significantly higher correspondence between time series of frontal brain activity before and after SBF attenuation than PC1 ($p < .001$, W(250) = 7.82, r = .49), PC123 ($p < .001$, W(250) = 7.82, r = .49), as well as IC1 ($p < .001$, W(250) = 7.82, r = .49). This was followed by significantly better performance by PC1 in contrast with PC123 ($p < .001$, W(250) = 7.82, r = .49) and IC1 ($p < .001$, W(250) = 7.82, r = .49). Lastly, PC123 was significantly more effective than IC1 (p = $p < .001$, W(250) = 7.82, r = .49). Figure 1, subplot C4, shows these results.

### 1.1.5 Criterion 2.5: $H(X|X') \leq H(X')$

Pairwise Wilcoxon rank sum suggested that all components preserved information content of frontal activity (PC1: $p < .001$ W(250) = 7.80, $M_{H(X|X')}$ = 2.63, $SD_{H(X|X')}$ = 1.64, $M_{H(X')}$ = 11.89, $SD_{H(X')}$ = 5.73, r = .49, PC12: $p < .001$ W(250) = 7.81, $M_{H(X|X')}$ = 3.71, $SD_{H(X|X')}$ = 1.63, $M_{H(X')}$ = 16.87, $SD_{H(X')}$ = 7.54, r = .49, PC123: $p < .001$ W(250) = 7.74, $M_{H(X|X')}$ = 3.81, $SD_{H(X|X')}$ = 1.65, $M_{H(X')}$ = 9.64, $SD_{H(X')}$ = 4.23, r = .48, IC1: $p < .001$, W(250) = 5.37, $M_{H(X|X')}$ = 4.84, $SD_{H(X|X')}$ = 1.63, $M_{H(X')}$ = 6.38, $SD_{H(X')}$ = 1.82, r = .34). Figure 1, subplot C5, depicts these results.
Kruskal-Wallis test indicated a significant effect of choice of component on preservation of information content of NIRS time series (i.e., $H(X') - H(X|X') \geq 0$) ($p < .001$, H(3, 503) = 65.70, r = .36). Post hoc paired comparison implied a significantly higher preservation of information content of frontal brain activity with respect to PC12 in comparison with PC1 ($p < .001$, W(250) = 7.82, $M_{PC12}$ = 13.04, $SD_{PC12}$ = 8.42, $M_{PC1}$ = 9.25, $SD_{PC1}$ = 6.47), PC123 ($p < .001$, r = .49, W(250) = 7.19, $M_{PC123}$ = 5.85, $SD_{PC123}$ = 4.86, r = .47), as well as IC1 ($p < .001$, W(250) = 7.82, $M_{IC1}$ = 1.53, $SD_{IC1}$ = 2.15, r = .49). Furthermore, PC1 was significantly more effective than PC123 ($p < .001$, W(250) = 6.46, r = .41) and IC1 ($p < .001$, W(250) = 7.82, r = .49). Lastly, we found PC123 significantly better than IC1 ($p < .001$, W(250) = 7.81, r = .49). Figure 2 shows these results.

## 1.2 CTE

Wilcoxon rank sum test implied significant effect of SBF on NIRS time series of frontal brain activity of participants during conversation ($p < .001$, W(17) = 3.73, M = .13, SD = .13). In what follows, we examine the effectiveness of PCA- and ICA-based SBF attenuation algorithms on reduction of observed impact of SBF on NIRS frontal brain activity as well as their utility in preservation of information content of this activity in resulting NIRS time series through investigation of Criterion 2.1 through Criterion 2.2.

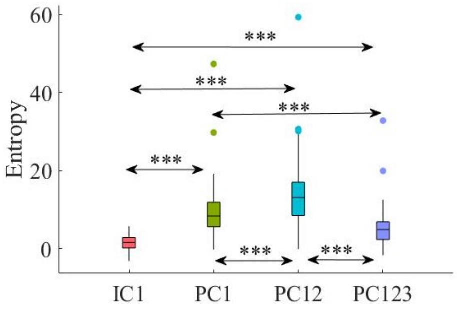


**Figure 2.** Comparison of maximal preservation of information content of frontal brain activity (i.e., $H(X') - H(X|X') \geq 0$, Criterion 2.5) after application of PCA- and ICA-based SBF attenuation algorithms in Verbal Fluency Task (VFT). Asterisks mark the components with significant difference (*: p < .05, **: p < .01, ***: p < .001).

### 1.2.1 Criterion 2.1: $TE(Y \rightarrow X') \leq TE(Y \rightarrow X)$

Kruskal-Wallis implied a significant effect of choice of components (p < .001, H(4, 89) = 29.66, r = .58). In addition, post hoc comparison suggested that all components significantly reduced SBF effect in comparison with $TE(Y \rightarrow X)$ (PC1: p < .001, W(34) = 3.72, M = .011, SD = .03, r = .62, PC12: p < .001, W(34) = 3.72, M = .009, SD = .02, r = .62, PC123: p < .001, W(34) = 3.72, M = .02, SD = .03, r = .62, IC1: < .001, W(34) = 3.72, M = .02, SD = .03, r = .62). On the other hand, whereas this comparison indicated non-significant difference between PC1 and PC12 (p = .95, W(34) = .03, r = .01) as well as PC123 and IC1 (p = .65, W(34) = .46, r = .08), we found significant differences in reduction of TE between PC1 and PC123 (p < .001, W(34) = 3.42, r = .57), PC1 and IC1(p < .001, W(34) = 3.68, r = .61), PC12

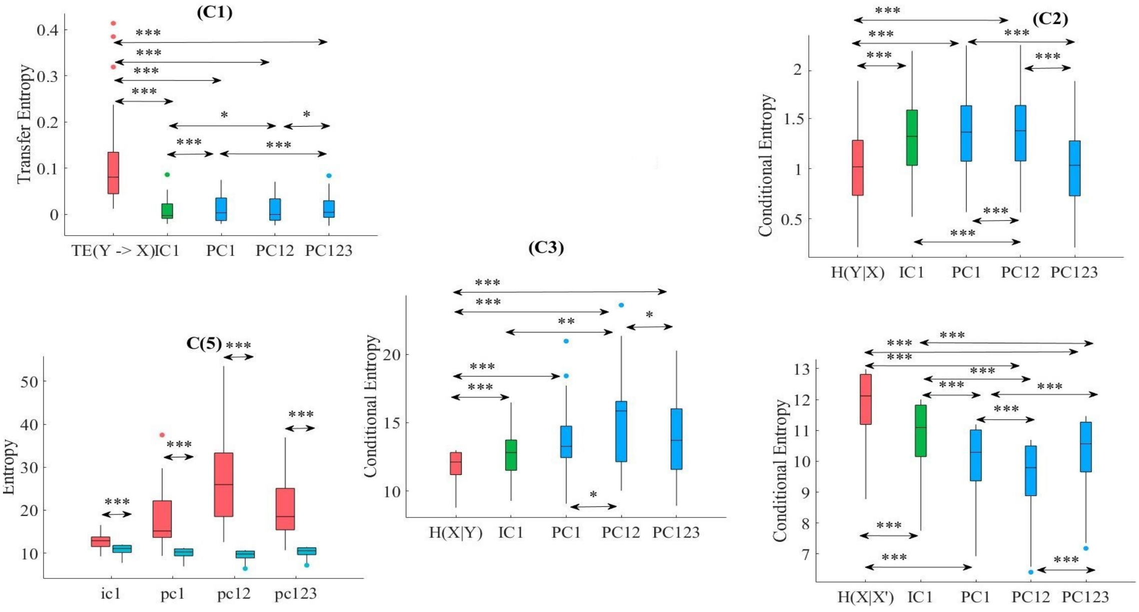


**Figure 3.** CTE - C1: Criterion 2.1: $TE(Y \rightarrow X') \leq TE(Y \rightarrow X)$, C2: Criterion 2.2: $H(Y|X) \leq H(Y|X')$, C3: Criterion 2.3: $H(X|Y) \leq H(X'|Y)$, C4: Criterion 2.4: $H(X|X') \leq H(X|Y)$, C5: Criterion 2.5: $H(X|X') \leq H(X')$. In these subplots, IC1, PC1, PC12, and PC123 refer to the measured quantity by these components after their respective SBF attenuation algorithms. Asterisks mark the components with significant difference (*: p < .05, **: p < .01, ***: p < .001).

and PC123 ($p < .05$, W(34) = 2.11, r = .35), as well as PC12 and IC1 ($p < .03$, W(34) = 2.55, r = .43). Figure 3, subplot C1, shows these results.

### 1.2.2 Criterion 2.2: $H(Y|X) \leq H(Y|X')$

Kruskal-Wallis implied a significant effect of choice of components ($p < .05$, H(4, 89) = 10.54, r = .34). Post hoc comparison implied a significant reduction of SBF-related information in frontal brain activity of participants by PC1 ($p < .001$, W(34) = 3.72, $M_{H(Y|X)}$ = 1.03, $SD_{H(Y|X)}$ = .47, $M_{PC1}$ = 1.32 $SD_{PC1}$ = .47, r = .62), PC12 ($p < .001$, W(34) = 3.72, $M_{PC12}$ = 1.32, $SD_{PC12}$ = .48, r = .69), and IC1 ($p < .001$, W(34) = 3.72, $M_{IC1}$ = 1.27, $SD_{IC1}$ = .47, r = .69). However, it indicated non-significant with respect to PC123 (p = .29, W(34) = 1.07, $M_{PC123}$ = 1.03, $SD_{PC123}$ = .47, r = .18). Moreover, this comparison indicated that adaptation of PC1 resulted in a significantly more reduced SBF-related information than PC123 ($p < .001$, W(34) = 3.72, r = .69) as well as IC1 ($p < .001$, W(34) = 3.72, r = .69). Similarly, we found PC12 significantly more effective than PC123 ($p < .001$, W(34) = 3.72, r = .69) and IC1 ($p < .001$, W(34) = 3.72, r = .69). Additionally, difference between IC1 and PC123 was significant ($p < .001$, W(34) = 3.72, r = .69). However, we found non-significant difference between PC1 and PC12 (p = .45, W(34) = .76, r = .13). Figure 3, subplot C2, shows these results.

### 1.2.3 Criterion 2.3: $H(X|Y) \leq H(X'|Y)$

We observed a significant effect of choice of components in resulting NIRS data ($p < .01$, H(4, 89) = 16.47, r = .51). Post hoc comparison suggested that all components were significantly effective in reducing the degree of correspondence between SBF and NIRS time series of frontal brain activity (PC1: $p < .001$, W(34) = 3.72, $M_{(X|Y)}$ = 11.58, $SD_{(X|Y)}$ = 1.53, $M_{PC1}$ = 13.76, $SD_{PC1}$ = 3.00, r = .69, PC12: $p < .001$, W(34) = 3.72, $M_{PC12}$ = 15.44, $SD_{PC12}$ = 3.83, r = .69, PC123: $p < .001$, W(34) = 3.72, $M_{PC123}$ = 14.07, $SD_{PC123}$ = 3.11, r = .69, IC1: $p < .001$, W(34) = 3.72, $M_{IC1}$ = 12.81, $SD_{IC1}$ = 1.98, r = .69). Additionally, this comparison implied that adaptation of PC12 yielded a significantly better performance than PC1 ($p < .03$, W(34) = 2.19, r = .37), PC123 ($p < .03$, W(34) = 2.46, r = .41), as well as IC1 ($p < .01$, W(34) = 2.90, r = .48). However, we found non-significant difference between PC1 and PC123 (p = .74, W(34) = .33, r = .06), PC1 and IC1 (p = .20, W(34) = 1.28, r = .21), as well as PC123 and IC1 (p = .17, W(34) = 1.42, r = .24). Figure 3, subplot C3, shows these results.

### 1.2.4 Criterion 2.4: $H(X|X') \leq H(X|Y)$

Kruskal-Wallis indicated significant effect of choice of components ($p < .001$, H(4, 89) = 25.08, r = .53). Post hoc comparison suggested that all components were significantly effective in retaining the correspondence between time series of frontal activity before and after application of SBF attenuation algorithms in comparison with SBF (PC1: $p < .001$, W(34) = 3.72, $M_H(X|Y)$ = 11.58, $SD_H(X|Y)$ = 1.53, $M_{PC1}$ = 10.30, $SD_{PC1}$ = 1.56, r = .69, PC12: $p < .001$, W(34) = 3.72, $M_{PC12}$ = 9.12, $SD_{PC12}$ = 1.55, r = .69, PC123: $p < .001$, W(34) = 3.72, $M_{PC123}$ = 9.90, $SD_{PC123}$ = 1.55, r = .69, IC1: $p < .001$, W(34) = 3.72, $M_{IC1}$ = 10.57, $SD_{IC1}$ = 1.55, r = .69). Moreover, this comparison implied that PC12 resulted in significantly higher correspondence between time series of frontal brain activity before and after SBF attenuation than PC1 ($p < .001$, W(34) = 3.72, r = .69), PC123 ($p < .001$, W(34) = 4.24, r = .71), as well as IC1 ($p < .001$, W(34) = 3.72, r = .69). This was followed by significantly better performance by PC1 in contrast with PC123 ($p < .001$, W(34) = 3.72, r = .69) and IC1 ($p < .001$, W(34) = 3.72, r = .69). Lastly, PC123 was significantly more effective than IC1 (p = $p < .001$, W(34) = 3.72, r = .69). Figure 3, subplot C4, shows these results.

### 1.2.5 Criterion 2.5: $\boldsymbol{H(X|X') \leq H(X')}$

Pairwise Wilcoxon rank sum suggested that all components preserved information content of frontal activity (PC1: p $<$ .001, W(34) = 3.72, M$_{H(X|X')}$ = 10.30, SD$_{H(X|X')}$ = 1.56, M$_{H(X')}$ = 18.17, SD$_{H(X')}$ = 7.48, r = .69, PC12: p $<$ .001, W(34) = 3.72, M$_{H(X|X')}$ = 9.12, SD$_{H(X|X')}$ = 1.55, M$_{H(X')}$ = 27.68, SD$_{H(X')}$ = 11.71, r = .69 PC123: p $<$ .001, W(34) = -3.72, M$_{H(X|X')}$ = 10.18, SD$_{H(X|X')}$ = 1.55, M$_{H(X')}$ = 20.58, SD$_{H(X')}$ = 7.24, r = .88, IC1: p $<$ .001, W(34) = 3.72, M$_{H(X|X')}$ = 10.57, SD$_{H(X|X')}$ = 1.55 M$_{H(X')}$ = 12.84, SD$_{H(X')}$ = 2.00, r = .69). Figure 3, subplot C5, depicts these results.
Kruskal-Wallis test indicated a significant effect of choice of component on preservation of information content of NIRS time series (i.e., $H(X') - H(X|X') \geq 0$) (p $<$ .01, H(3, 71) = 13.16, r = .43). Post hoc comparison implied significantly higher preservation of information content of frontal brain activity with respect to PC12 in comparison with PC1 (p $<$ .001, W(34) = 3.54, M$_{PC12}$ = 18.53, SD$_{PC12}$ = 10.81, M$_{PC1}$ = 7.87, SD$_{PC1}$ = 7.04), r = .59, PC123 (p $<$ .001, W(34) = 3.72, M$_{PC123}$ = 10.68, SD$_{PC123}$ = 6.45, r = .62), as well as IC1 (p $<$ .001, W(34) = 3.72, M$_{IC1}$ = 2.27, SD$_{IC1}$ = 1.20, , r = .62). Furthermore, PC1 was significantly more effective than IC1 (p $<$ .01, W(34) = 3.03, r = .21). Lastly, we found PC123 significantly better than IC1 ($<$ .001, W(34) = 3.72, r = .23). However, this test implied that difference between PC1 and PC123 was non-significant (p = .07, W(34) = 1.85, r = .16). Figure 4 shows these results.

## 1.3 LMT

Wilcoxon rank sum test implied significant effect of SBF on NIRS time series of frontal brain activity of participants while performing VFT working memory task (p $<$ .001, W(25) = 4.46, M = .58, SD = .19, r = .70). In what follows, we examine the effectiveness of PCA- and ICA-based SBF attenuation algorithms on reduction of observed impact of SBF on NIRS frontal brain activity as well as their utility in preservation of information content of this activity in resulting NIRS time series through investigation of Criterion 2.1 through Criterion 2.5.

### 1.3.1 Criterion 2.1: $\boldsymbol{TE(Y \rightarrow X') \leq TE(Y \rightarrow X)}$

Kruskal-Wallis test implied significant effect of choice of components (p $<$ .001, H(4, 129) = 56.39, r = .66). Moreover, post hoc comparison implied that all components significantly reduced SBF effect in comparison with $TE(Y \rightarrow X)$ (PC1: p $<$ .001, W(50) = 4.46, M = .16, SD= .15, r = .62, PC12: p $<$ .001, W(50) = 4.43, M = .08, SD = .13, r = .61, PC123: p $<$ .001, W(50) = 4.46, M = .18, SD = .16, r = .62, IC1:

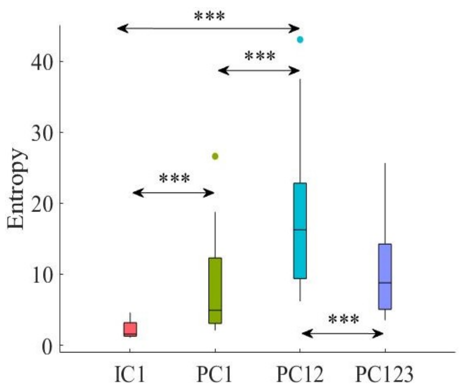


**Figure 4.** Comparison of maximal preservation of information content of frontal brain activity (i.e., $H(X') - H(X|X') \geq 0$, Criterion 2.5) after application of PCA- and ICA-based SBF attenuation algorithms in Conversational Task Experiment (CTE). Asterisks mark the components with significant difference (*: p $<$ .05, **: p $<$ .01, ***: p $<$ .001).

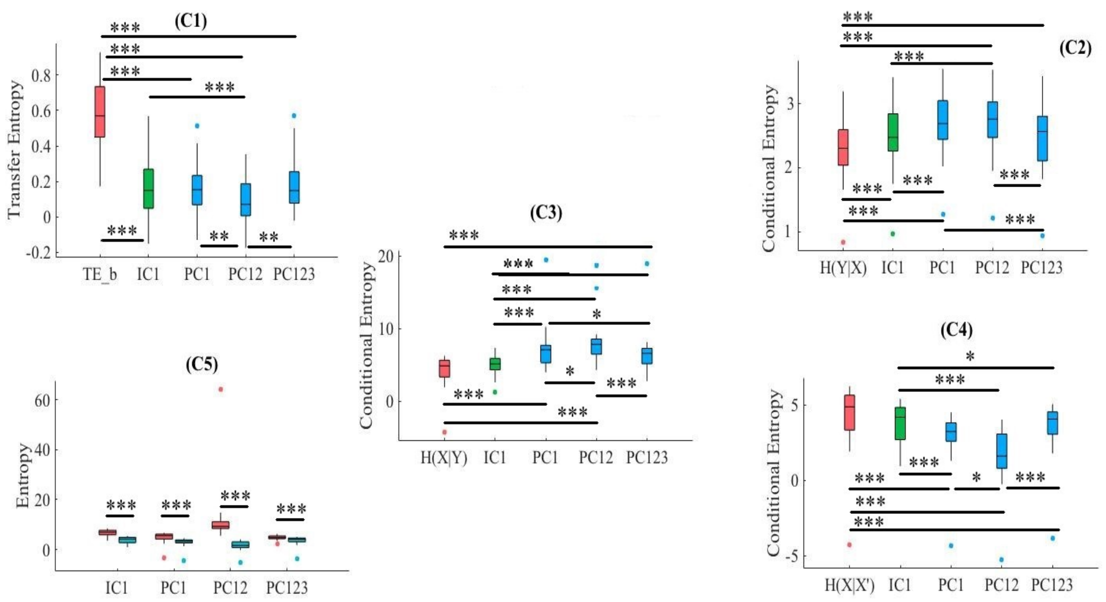


**Figure 5.** LMT. C1: Criterion 2.1: $TE(Y \rightarrow X') \leq TE(Y \rightarrow X)$, C2: Criterion 2.2: $H(Y|X) \leq H(Y|X')$, C3: Criterion 2.3: $H(X|Y) \leq H(X'|Y)$, C4: Criterion 2.4: $H(X|X') \leq H(X|Y)$, C5: Criterion 2.5: $H(X|X') \leq H(X')$. In these subplots, IC1, PC1, PC12, and PC123 refer to the measured quantity by these components after their respective SBF attenuation algorithms. Asterisks mark the components with significant difference (*: p < .05, **: p < .01, ***: p < .001).

p < .001, W(50) = 4.43, M = .18, SD = .17, r = .61). Post hoc comparison indicated that PC12 significantly performed better in attenuation of the effect of SBF than PC1 (p < .01, W(50) = 2.73, r = .38), PC123 (p < .01, W(50) = 3.04, r = .42), and IC1 (p < .001, W(50) = 3.34, r = .46). On the other hand, we observed non-significant differences between PC1 and PC123 (p = .36, W(50) = .93, r = .13), PC1 and IC1 (p = .47, W(50) = .72, r = .10), as well as PC123, and IC1 (p = .75, W(50) = .32, r = .04). Figure 5, subplot C1, illustrates these results.

### 1.3.2 Criterion 2.2: $\boldsymbol{H(Y|X) \leq H(Y|X')}$

Kruskal-Wallis implied a significant effect of choice of components (p < .01, H(4, 129) = 13.32, r = .32). Post hoc comparison indicated a significant reduction of SBF-related information in frontal brain activity of participants by PC1 (p < .001, W(50) = 4.46, $M_{H(Y|X)}$ = 2.31, $SD_{H(Y|X)}$ = .50, $M_{PC1}$ = 2.69, $SD_{PC1}$= .48, r = .62), PC12 (p < .001, W(50) = 4.46, $M_{PC12}$ = 2.71, $SD_{PC12}$ = .50, r = .62), PC123 (p < .001, W(50) = 3.95, $M_{PC123}$ = 2.47, $SD_{PC123}$ = .53, r = .55), and IC1 (p < .001, W(50) = 4.46, $M_{IC1}$ = 2.49, $SD_{IC1}$ = .53, r = .62). Additionally, this comparison indicated that adaptation of PC1 resulted in a significantly more reduced SBF-related information than PC123 (p < .001, W(50) = 4.23, r = .49) as well as IC1 (p < .001, W(50) = 4.33, r = .49). Similarly, we found PC12 significantly more effective than PC123 (p < .001, W(50) = 4.10, r = .49) and IC1 (p < .001, W(50) = 4.36, r = .49). However, we found non-significant differences between PC1 and PC12 (p = .49, W(50) = .70, r = .10) as well as IC1 and PC123 was significant (p = .14, W(50) = 1.49, r = .21). Figure 5, subplot C2, shows these results.

### 1.3.3 Criterion 2.3: $\boldsymbol{H(X|Y) \leq H(X'|Y)}$

Kruskal-Wallis indicated a significant effect of choice of components in resulting NIRS data (p < .001, H(4, 129) = 47.41, r = .61). Post hoc comparison suggested that all components were significantly effective in reducing the degree of correspondence between SBF and NIRS time series of frontal brain activity (PC1:

$p < .001$, W(50) = 4.46, $M_{H(X|Y)}$ = 4.22, $SD_{H(X|Y)}$ = 2.21, $M_{PC1}$ = 7.11, $SD_{PC1}$ = 3.03, r = .62, PC12: $p < .001$, W(50) = 4.46, $M_{PC12}$ = 8.17, $SD_{PC12}$ = 3.01, r = .62, PC123: $p < .001$, W(50) = 4.46, $M_{PC123}$ = 6.57, $SD_{PC123}$ = 2.96, r = .62). However, we observed that the effect of IC1 was non-significant (IC1: p = .10, W(50) = 1.66, $M_{IC1}$ = 5.09, $SD_{IC1}$ = 1.36, r = .23). In addition, this comparison indicated that adaptation of PC12 yielded a significantly better performance than PC1 ($p < .03$, W(50) = 2.30, r = .32), PC123 ($p < .001$, W(50) = 3.42, r = .47), as well as IC1 ($p < .001$, W(50) = 4.46, r = .62). Moreover, PC1 was significantly more effective than PC123 ($p < .05$, W(50) = 1.97, r = .27) and IC1 ($p < .001$, W(50) = 3.95, r = .55). Additionally, PC123 was significantly different from IC1 ($p < .001$, W(50) = 2.58, r = .36). Figure 5, subplot C3, plots these results.

### 1.3.4 Criterion 2.4: $H(X|X') \leq H(X|Y)$

Kruskal-Wallis indicated a significant effect of choice of components ($p < .001$, H(4, 129) = 38.04, r = .54). Post hoc comparison suggested that all components were significantly effective in retaining the correspondence between time series of frontal activity before and after application of SBF attenuation (PC1: $p < .01$, W(50) = 3.09, $M_{H(X|Y)}$ = 4.22, $SD_{H(X|Y)}$ = 2.22, $M_{PC1}$ = 2.88, $SD_{PC1}$ = 1.72, r = .43, PC12: $p < .001$, W(50) = 4.46, $M_{PC12}$ = 1.62, $SD_{PC12}$ = 1.88, r = .62, PC123: $p < .03$, W(50) = 3.30, $M_{PC123}$ = 3.52, $SD_{PC123}$ = 1.74, r = .46) However, we observed that the effect of IC1 was non-significant (p = .13, W(50) = 1.51, $M_{IC1}$ = 3.67, $SD_{IC1}$ = 1.47, r = .21). Additionally, this comparison implied that PC12 resulted in significantly higher correspondence between time series of frontal brain activity before and after SBF attenuation than PC1 ($p < .03$, W(50) = 2.98, r = .41), PC123 ($p < .001$, W(50) = 3.80, r = .53), as well as IC1 ($p < .001$, W(50) = 3.95, r = .55). Although PC1 performed significantly better than PC123 ($p < .001$, W(50) = 4.40, r = .61), its difference with IC1 was non-significant (p = .10, W(50) = 1.66, r = .23). Lastly, difference between PC123 and IC1 was no-significant more effective than IC1 (p = .89, W(50) = .14, r = .02). Figure 5, subplot C4, shows these results.

### 1.3.5 Criterion 2.5: $H(X|X') \leq H(X')$

Pairwise Wilcoxon rank sum suggested that all components preserved information content of frontal activity (PC1: $p < .001$ W(50) = 3.64, $M_{H(X|X')}$ = 2.88, $SD_{H(X|X')}$ = 1.72, $M_{H(X')}$ = 4.80, $SD_{H(X')}$ = 2.11, r = .50, PC12: $p < .001$, W(50) = 4.46, $M_{H(X|X')}$ = 1.62, $SD_{H(X|X')}$ = 1.88, $M_{H(X')}$ = 11.55, $SD_{H(X')}$ = 10.94, r = .62, PC123: $p < .001$, W(50) = 3.67, $M_{H(X|X')}$ = 3.53, $SD_{H(X|X')}$ = 1.74, $M_{H(X')}$ = 4.83, $SD_{H(X')}$ = .91, r = .51, IC1: $p < .001$, W(50) = 4.46, $M_{H(X|X')}$ = 3.67, $SD_{H(X|X')}$ = 1.47, $M_{H(X')}$ = 6.67, $SD_{H(X')}$ = 1.40, r = .62). Figure 5, subplot C5, depicts these results.
Kruskal-Wallis test indicated a significant effect of choice of component on preservation of information content of NIRS time series (i.e., $H(X') - H(X|X') \geq 0$) ($p < .01$, H(3, 103) = 28.08, r = .27). Post hoc paired comparison implied a significantly higher preservation of information content of frontal brain activity with respect to PC12 in comparison with PC1 ($p < .001$, W(50) = 4.38, $M_{PC1}$ = 1.91, $SD_{PC1}$ = 2.46, $M_{PC12}$ = 9.92, $SD_{PC12}$ = 11.29, r = .61), PC123 ($p < .001$, W(50) = 4.46, $M_{PC123}$ = 1.32, $SD_{PC123}$ = 1.77, r = .61), as well as IC1 ($p < .001$, W(50) = 4.46, $M_{IC1}$ = 2.30, $SD_{IC1}$ = .51, r = .62). IC1 was significantly more effective than and PC1 ($p < .03$, W(50) = 2.50, r = .35) and PC123 ($p < .01$, W(50) = 3.80, r = .53). Last, we found that PC1 was significantly more effective than PC123 ($p < .03$, W(50) = 2.45, r = .34). Figure 6 shows these results.

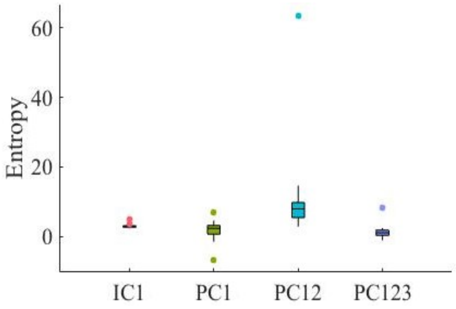


**Figure 6.** Comparison of maximal preservation of information content of frontal brain activity (i.e., $H(X') - H(X|X') \geq 0$, Criterion 2.5) after application of PCA- and ICA-based SBF attenuation algorithms in Verbal Fluency Task (VFT). Asterisks mark the components with significant difference (*: p $<$ .05, **: p $<$ .01, ***: p $<$ .001).
